# Ultrafast Capture of Per- and Polyfluoroalkyl Substances from Water by Mesoporous Zirconium Metal–Organic Frameworks

**DOI:** 10.1021/acsmaterialslett.6c00186

**Published:** 2026-04-24

**Authors:** Sergio Marugán-Benito, Rodrigo Gil-San-Millan, María Alarcón del Río, Lutz Ahrens, Edward Loukopoulos, Ana E. Platero-Prats

**Affiliations:** † Departamento de Química Inorgánica, Facultad de Ciencias, 16722 Universidad Autónoma de Madrid, Campus de Cantoblanco, 28049 Madrid, Spain; ‡ Department of Aquatic Sciences and Assessment, 8095 Swedish University of Agricultural Sciences (SLU), Uppsala SE-75007, Sweden; § Condensed Matter Physics Center (IFIMAC), Universidad Autónoma de Madrid, Campus de Cantoblanco, 28049 Madrid, Spain; ∥ Instituto de Catálisis y Petroleoquímica (ICP-CSIC), c/Marie Curie 2, Madrid 28049, Spain

## Abstract

Contamination of surface and groundwater sources by emerging persistent pollutants has presented a global environmental challenge that demands advanced remediation materials. This work exploits the large mesopores and unsaturated inorganic nodes in MIP-206-based metal–organic frameworks (MOFs) for the highly efficient adsorption of perfluorocarboxylic acids (PFCAs) from water. The materials display excellent performance for long-chain PFCAs, achieving removal efficiencies up to >99% within seconds. Detailed mechanistic studies, including synchrotron analyses, provide key insights into the development of optimized PFCA sorbents via multiple interaction types.

## Graphical abstract

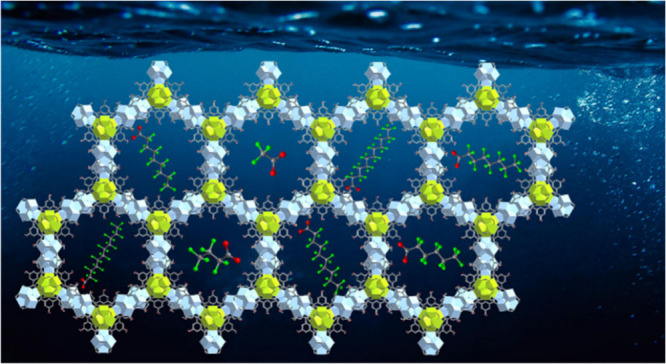

The rise of per- and polyfluoroalkyl substances (PFAS) as persistent water contaminants has been recognized as a major environmental challenge. Decades of extensive PFAS use in industrial production, combined with the extreme stability of their C–F bonds, has led to substantial bioaccumulation and toxicity in aquatic systems worldwide.^1^ Among these compounds, perfluoroalkyl carboxylic acids (PFCAs, Figure 1) have received major attention and regulatory scrutiny,^2^ due to their high degradation resistance in both natural environments and conventional treatment processes.^3,4^

**Figure 1:**
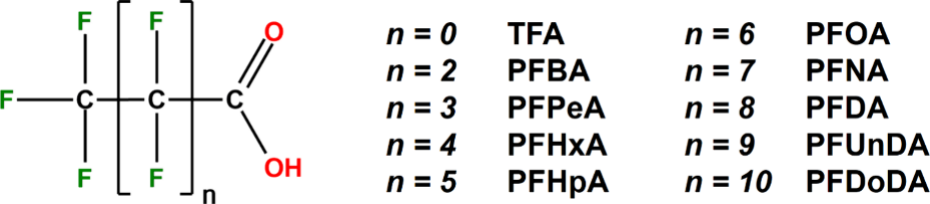
General PFCA structure and the corresponding analogues studied in this work.

The limited success of conventional PFCA remediation methods has prompted a search for alternative technologies, with adsorption emerging as the most promising approach.^5^ In this context, metal–organic frameworks (MOFs) have attracted significant attention as advanced adsorbents due to their high surface areas, high stability and chemical tunability.^6^ Such characteristics are especially important in PFCA treatment, unlocking opportunities to capture these pollutants not only through pore filling but also via chemical interactions^7^ and formation of strong bonds.^8^ Recent studies have started to explore these principles for PFCA sorption with encouraging results.^9^ Nevertheless, key challenges remain, including slow adsorption kinetics, limited exploration of operational parameters, use of MOFs with low synthetic scalability, and a generally incomplete understanding of the exact interplay between adsorbent and contaminant.^9,10^ Considering the distinct chemistry of PFCAs, as well as the vast diversity of available MOFs, rational material selection and mechanistic insights are crucial for the development of truly advanced sorbents.

In this study, we have identified MIP-206 and MIP-206-OH, two isostructural mesoporous frameworks based on Zr_6_O_8_/Zr_12_O_22_ nodes (Figure 2A-B), as suitable platforms for the capture of PFCA pollutants in water via a multifaceted adsorption process. Beyond mesopore filling, mechanistic investigations reveal the preferential coordination of PFCAs to the unsaturated Zr_6_O_8_ nodes as the dominant adsorption mechanism. Under certain conditions, additional weak forces, including hydrophobic interactions and hydrogen bonding, further contribute to retention. These favorable environments lead to exceptional adsorption performance for long-chain PFCAs, with excellent removal efficiencies across a wide range of pollutant concentrations and ultrafast adsorption kinetics in comparison to MOFs reported to date. Beyond the overall performance, these findings mark an important step toward universal PFAS sorbents, providing deeper mechanistic insight into MOF/PFAS interactions and guiding the rational design of next-generation materials.

**Figure 2:**
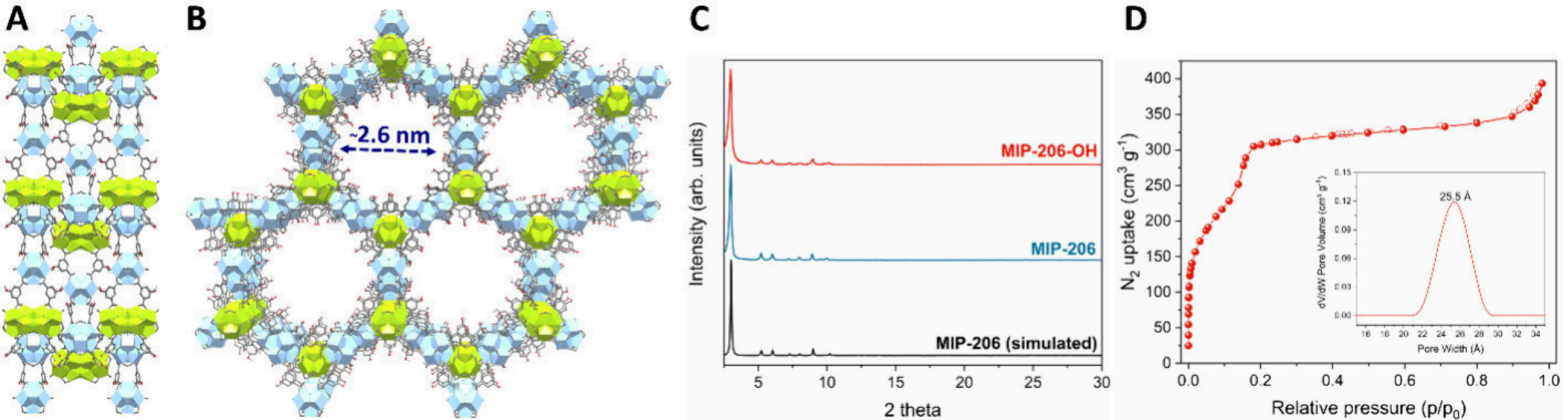
(A-B) View of the MIP-206 framework (−OH analogue) depicting (A) 2D layers forming along the *ac* plane and (B) 1D mesoporous cavities along the *c* axis. The two different types of inorganic clusters (Zr_6_ and Zr_12_) are denoted as light blue and light green polyhedra, respectively. (C) PXRD patterns of the isostructural as-made MOFs, compared to the one calculated from the crystal structure of MIP-206. (D) N_2_ sorption isotherm of MIP-206-OH recorded at 77 K. The inset shows the corresponding pore size distribution curve. Corresponding data for MIP-206 can be found in the ESI.

The MOF materials of this work were selected due to their inherent structural features, which enable opportunities to maximize PFAS capture via multiple adsorption mechanisms: the available mesopores of up to 2.6 nm in diameter (Figure 2B) can accommodate short- and long-chain pollutant analogues in large quantities. Moreover, the difference in functional group between these materials (−H versus −OH) offers an ideal system to probe how pore hydrophilicity influences PFAS capture. Beyond physical adsorption, the highly acidic carboxylate group in PFCAs can strongly bind to open metal sites of MOFs based on Zr_6_O_8_ nodes such as the ones used in this study.

Both MIP-206 and MIP-206-OH were synthesized at gram scale under solvothermal conditions at 180 °C using formic acid as the reaction solvent following previously reported methods^11,12^ (see the Supporting Information), yielding highly microcrystalline solids (Figures S1–S2). It should be noted that the above studies have also reported synthetic protocols on a 10 g scale, albeit under the same harsh solvothermal conditions. Subsequent powder X-ray diffraction (PXRD), Fourier-transform infrared spectroscopy (FT-IR) and gas adsorption measurements confirmed structural features (Figure 2C–D and Section S2 in the Supporting Information) consistent with those reported for MIP-206 and MIP-206-OH. Textural analysis from N_2_ isotherms at 77 K verified the presence of the expected mesopores (2.3–2.6 nm in size) within both MOFs. While the main framework compositions matched well to previous reports, ^1^H-Nuclear Magnetic Resonance (NMR) analysis revealed a negligible amount of capping formate species at the zirconium nodes for both materials under multiple MOF digestion protocols (Figures S11–S14), contrasting with the DFT-simulated crystallographic structures originally reported.^12^ To account for this discrepancy, one ^–^OH/H_2_O pair per formate was considered in the final chemical formulas to maintain the charge balance. Thermogravimetric analysis (TGA, Figures S16–S17) further supported these adjustments.

The PFAS adsorption performance of MIP-206 and MIP-206-OH was first evaluated by immersing each MOF (dosage of 1.0 mg·mL^–1^) in aqueous PFCA solutions at room temperature for 24 h (SI, Section S3). The starting and final PFCA concentrations were quantified by ^19^F-NMR spectroscopy. Preliminary studies focused on perfluorooctanoic acid (PFOA), considering its higher environmental relevance and regulatory priority in comparison with other PFCAs. Moreover, the MOF material DUT-67 ^13^ was also synthesized (Sections S1–S2, Supporting Information) and evaluated for PFCA adsorption under the same conditions, as a mechanistically analogous control material. Similar to the MIP-206 family, DUT-67 also features unsaturated Zr_6_O_8_ nodes^14^ and shows high stability in aqueous or acidic environments; however, its framework is microporous instead of mesoporous (Figure S10). The corresponding PFCA capture data thus provide a suitable basis to evaluate the influence of micro- versus mesoporosity, allowing us to distinguish the effect of physical adsorption from other chemical interactions, such as coordination bonding.

Both MIP-206 and MIP-206-OH exhibited excellent removal efficiencies across a wide range of pollutant concentrations at parts per million levels (Figure 3A, Table S3), with no detectable PFOA signals for initial solutions of 50 and 100 ppm. Time-dependent experiments under these conditions showed ultrafast adsorption kinetics, as equilibrium is reached within 30 s (Figure 3B, Table S4). To the best of our knowledge, this equilibrium time is among the shortest reported for PFAS sorption in MOFs, comparable only to that of the mesoporous NU-1000 framework (t_eq_ = 1 min).^15^ In contrast, most MOFs reported to date require several hours to reach equilibrium (Table S5).^10^ Although kinetic modeling could not be accurately performed due to the extremely rapid adsorption, PFAS uptake in MOFs typically follows a pseudo-second order kinetic model,^10^ in line with the formation of strong surface interactions between the MOF and the pollutant. Notably, while MIP-206 showed a decline in performance at higher PFOA concentrations (93% and 27% capture at 500 and 1000 ppm, respectively, Tables S3, S7–S8), MIP-206-OH maintained >99% removal efficiency even at 1000 ppm, underscoring its highly favorable pore environment for PFAS capture. In comparison, microporous DUT-67 also exhibited high PFOA removal efficiency at initial concentrations of 50 and 100 ppm (Table S3, Figure 3A), albeit with a significantly longer equilibrium time (only 45% captured after 30 min, Table S3). A pronounced decrease in performance compared to the MIP-206 family is also observed at higher PFOA levels (65% removal efficiency at 500 ppm of pollutant). These results highlight the critical role of mesoporosity in enabling rapid adsorption, particularly at elevated contaminant amounts.

**Figure 3:**
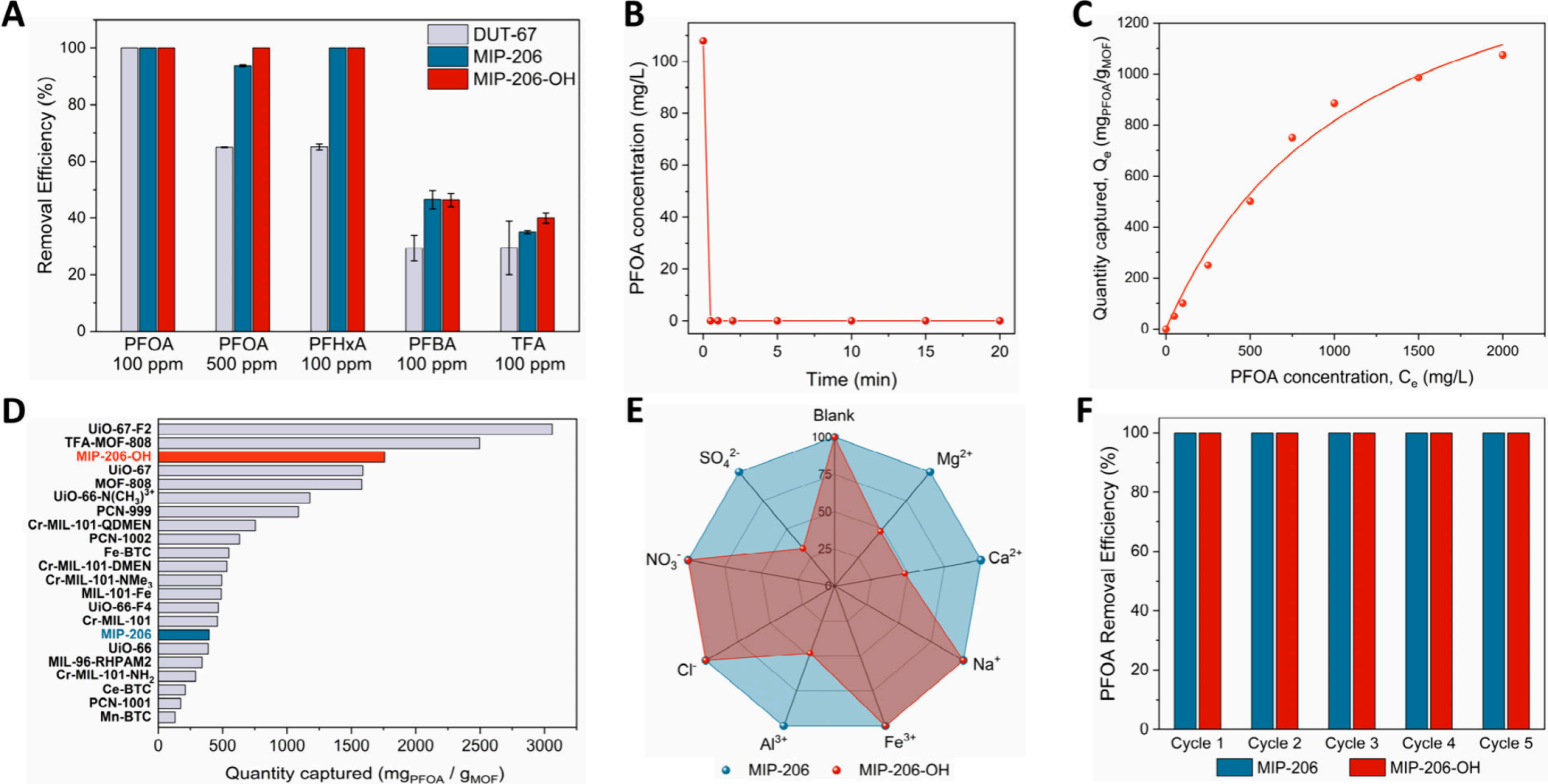
(A) Selected PFAS removal efficiencies for the microporous (DUT-67) and mesoporous (MIP-206 and MIP-206-OH) materials of this work at ppm levels. (B) PFOA adsorption studies for MIP-206-OH over time (C_0_ = 100 ppm). (C) PFOA adsorption capacity studies for MIP-206-OH, fitted with the Langmuir model. (D) PFOA adsorption capacity data of MIP-206 and MIP-206-OH in comparison to the highest-performing MOF materials reported in the literature. Numeric values can be found in the ESI, Table S6. (E-F) PFOA removal efficiencies (C_0_ = 100 ppm) of MIP-206 and MIP-206-OH (E) in the presence of various ionic species (2–10 mM). (F) after multiple cycles.

Further investigations on the PFOA capture capabilities of MIP-206 and MIP-206-OH were performed to evaluate adsorption capacity, recyclability, and selectivity (Figure 3C–F). The PFOA adsorption capacity for both materials was quantified from experimental data using pollutant concentrations up to 3000 ppm. The resulting isotherms were fitted with both Langmuir and Freundlich models, with the Langmuir model showing higher R^2^ correlation coefficients in each case (0.98 versus 0.88 in MIP-206, 0.99 versus 0.95 in MIP-206-OH) as shown in Figures S18–S19. For MIP-206, the experimental PFOA uptake appears to plateau beyond concentrations of 500 ppm, leading to a Langmuir-determined maximum adsorption capacity of 395 mg_PFOA_/g_MOF_. This value indicates a moderate PFOA capacity in comparison with other state-of-the-art MOFs (Table S6). In contrast, MIP-206-OH showed significantly different behavior at increased pollutant concentrations, continuing to adsorb PFOA at a high rate. For a 50–2000 ppm concentration range, the maximum adsorption capacity calculated by the Langmuir model was determined to be 1758 mg_PFOA_/g_MOF_, placing this material among the highest performing MOFs reported for this metric to the best of our knowledge (Figures 3C–D, Table S6). Beyond the adsorption capacity, cycling experiments were performed to assess the reusability of MIP-206 and MIP-206-OH. As shown in Figure 3F, PFOA removal efficiencies for both materials remained consistent over five subsequent adsorption cycles. Corresponding PXRD patterns recorded after the fifth cycle (Figure S20) confirmed that the structural stability of both materials was preserved, indicating their suitability for repeated operation in practical applications.

Additional studies were performed to evaluate the PFOA adsorption behavior of these materials in the presence of common coexisting ions typically found in aqueous environments. The interfering agents tested included Na^+^, Ca^2+^, Mg^2+^, Fe^3+^, Al^3+^, Cl^–^, NO_3_^–^ and SO_4_^2–^ species (Table S7). As shown in Figure 3E, MIP-206 was found to be highly selective toward PFOA, maintaining its removal efficiency in all cases. For MIP-206-OH, similar behavior was observed in the presence of Na^+^, Fe^3+^, Cl^–^ and NO_3_^–^. However, the remaining ions interfered significantly, leading to decreased PFOA removal efficiencies (approximately 50% when Al^3+^, Ca^2+^ or Mg^2+^ were present and 30% in the case of SO_4_^2–^). Considering the chemical nature and behavior of these species, these effects likely arise due to competitive interactions at binding sites of either the sorbent (e.g., competitive coordination to the inorganic nodes) or the adsorbate, given that PFOA is present mainly in the anionic form under these conditions. The overall results reveal distinct differences in the performance of MIP-206 and MIP-206-OH in water matrices with a higher chemical complexity. This further suggests that their distinct pore environments influence the spatial arrangement of PFOA molecules within the pores, in turn affecting adsorption performance. Overall, both materials demonstrate very promising PFOA adsorption performance in several key metrics including removal efficiency, kinetics, maximum capacity, recycling, and selectivity.

We next explored the influence of the PFCA chain length on sorption performance by extending the pollutant scope at the same concentration levels (Figure 3A, Table S3) to include trifluoroacetic acid (TFA), perfluorobutanoic acid (PFBA) and perfluorohexanoic acid (PFHxA). As expected, accessible porosity continues to play an important role, with the microporous DUT-67 consistently showing lower removal efficiencies across all tested pollutants. Regarding the mesoporous materials, both MIP-206 and MIP-206-OH retain excellent removal efficiencies (>99%) for PFHxA. In contrast, shorter-chain analogues (TFA and PFBA) exhibited markedly lower uptakes (approximately 35–50%). This behavior is consistent with their distinct physicochemical properties: the smaller molecular size and reduced fluorine content of short-chain PFCAs enhance their water solubility and mobility,^3^ thereby reducing adsorption affinity. Their lower acidity and weaker hydrophobic character further limit interactions that favor binding. In line with this interpretation, lower removal efficiencies of shorter-chain PFCAs in comparison to those of PFOA were also observed in the presence of competing ions (Figure S21). Notably, while both MOFs performed similarly at 100 ppm, MIP-206-OH again surpassed MIP-206 at higher PFBA concentrations (500 ppm, Table S3), mirroring the trend observed for PFOA. This indicates that the −OH groups oriented toward the mesopores contribute to adsorption regardless of PFCA chain length, likely through structure-directing hydrogen bonding.^16,17^ The effect, however, appears to be concentration-dependent, diminishing at lower pollutant levels.

To further test this hypothesis and assess the practical limitations of the system, additional adsorption experiments (Figure S22) were performed in water samples spiked with a broader range of PFCA contaminants from C_4_ to C_11_ PFCAs at much lower concentration levels (individual [PFCA] = 250 ppb).^2^ Quantification was performed using ultrahigh performance liquid chromatography with tandem mass spectrometry (UPLC-MS/MS) as described elsewhere.^18^ As expected, both MOFs showed poor to moderate adsorption of short-chain PFCAs, an effect further amplified at these trace concentration levels. In contrast, removal efficiencies improved considerably as the PFCA chain size increased. Use of MIP-206 as sorbent notably led to captures of 92% and 97% for perfluoroundecanoic acid (PFUnDA) and perfluorododecanoic acid (PFDoDA), respectively, demonstrating its strong affinity toward long-chain PFCAs. Interestingly, under these dilute conditions, MIP-206 outperformed its −OH analogue, reversing the trend observed at ppm scale. This behavior likely arises from the increased hydrophilicity of MIP-206-OH, which promotes stronger competition with water molecules.^19^ Consequently, some hydroxyl groups may preferentially form hydrogen bonds with water rather than with the limited PFCA carboxylates, partially blocking pore access and reducing the adsorption capacity. These findings underscore the importance of systematic evaluations across varying concentration regimes to assess the practical potential of MOFs for PFAS remediation more accurately. Overall, both frameworks display strong promise, particularly for removing long-chain PFCAs of global concern across a wide range of pollutant levels.

Thorough postadsorption characterization was performed for both MOFs to elucidate their adsorption behavior and identify the origins of their high performance. PXRD data (Figures S20 and S23) showed no notable alteration in the main frameworks or their crystallinity, irrespective of the contaminant type. Importantly, qualitative ^1^H-/^19^F-NMR spectra verified the presence of PFCA molecules within the solid samples even after repeated washing with water and acetone (Figure S24), indicating strong binding. Complementary FT-IR spectra (Figures S25–S26) revealed the presence of characteristic peaks for C–F stretching bands in the 1250–1100 cm^–1^ region,^20^ as well as the absence of any signals related to uncoordinated carboxylic acid species. Further confirmation of these PFCA···MOF interactions was gained via solid-state NMR spectroscopy studies for ^19^F and ^13^C, before and after capture of PFOA. The pure PFOA sample showed ^19^F chemical shifts at the regions of δ = −83.9, −121.1, −124 and −128.4 ppm, in good agreement with previous reports^21^ (Figures S27–S28). These characteristic signals also appear in the postcapture solids of both MIP-206 and MIP-206-OH; however, all ^19^F resonances show an upfield shift (δ = −85.3, −121.2, −124.9, −125.5 and −129.6 ppm), consistent with PFOA incorporation within the MOFs.^16^ Moreover, substantial overlap is observed for the resonances of the inner CF_2_ groups located closer to the carboxylate group (region of 120–126 ppm), suggesting additional interactions of these groups with the adsorbent.^21^ Similar NMR analyses of these solids for ^13^C reveals clear changes in the characteristic C=O resonance of the carboxylate group of the pollutant, signifying different local environments.^22^ As shown in Figures S29–S30, the relevant signal shifts from 165.4 ppm in the pure PFOA to 157.2 and 160.7 ppm upon adsorption to MIP-206 and MIP-206-OH, respectively. This result further reinforces a PFOA binding mechanism,^16^ also influenced by the different local electronic environments within the linker of each framework (−H versus −OH). Overall, these analyses provide strong indications that PFCA molecules coordinate to the Zr clusters within the MOFs, in line with previous reports on MOFs based on unsaturated metal units.^8,16,23^

Despite the structural complexity of the MIP-206 series, a detailed inspection of the framework (Figure S31) highlights specific sites likely responsible for PFCA coordination. Out of ten potential carboxylate-binding positions (six on the Zr_12_O_22_ node and four on Zr_6_O_8_), eight are directed toward the 2D layers of the structure and lie within 3–5 Å of adjacent linkers, sterically hindering access by bulky PFCAs. Consequently, only two sites (both on the Zr_6_O_8_ nodes and oriented toward the mesopores) remain accessible for coordination. In agreement, ^1^H NMR-derived composition analyses indicate the presence of 0.15–0.7 PFCA molecules per cluster in the washed solids (Tables S8–S10), depending on pollutant concentration and chain length. As shown in Table S11, these strongly bound species can be readily desorbed using mild treatments (e.g., HCl/MeOH or aqueous NaCl solutions), enabling full regeneration of the materials for subsequent adsorption cycles.

Given the difficulties in obtaining high-quality single-crystal data for MIP-206 structures,^12^ advanced synchrotron characterization was performed to further investigate local structural changes and confirm PFCA coordination within the Zr_6_O_8_ clusters. X-ray absorption spectroscopy (XAS) provided detailed insight into the Zr coordination environment before and after PFOA adsorption. X-ray absorption near edge structure (XANES) measurements at the Zr *K*-edge for MIP-206-OH showed no detectable changes in the geometry or first-shell coordination (Figure S32). In contrast, extended X-ray absorption fine structure (EXAFS, Figures S33–S34) revealed subtle variations in Zr–O and Zr···Zr distances (approximately 1.6 and 3.2 Å, respectively, before phase correction), consistent with minor framework adjustments upon PFOA binding. These results indicate that adsorption induces small bond distortions, while preserving the overall coordination sphere and structural integrity of the MOF.

Successive pair distribution function (PDF) analysis was applied to total X-ray scattering data collected for both frameworks pre- and postsorption of PFCA sorption. In the short-range region of all PDFs (Figures 4 and S35–S36), three main contributions at *ca*. 2.2, 3.5, and 5.0 Å correspond to Zr–O, Zr···Zr and Zr···Zr_axial_ distances within the Zr_6_O_8_ clusters.^24^ After PFCA capture, additional features appear, consistent with replacement of the initial −OH/H_2_O pairs by PFCA molecules. These changes in the local structure of the MOFs were isolated and highlighted via differential PDF (dPDF) analyses.^24^ Characteristic peaks for PFCA chains are observed at 1.3–1.6 and 2.6–2.9 Å, corresponding to C–F, C–O, C–C bonds and C···F, C···C, F···F interatomic distances.^8^ Further variations were observed in the zirconium coordination environment, reflecting interactions with PFCA, with small bond contractions/expansions at ca. 2.0 Å (Zr–O) and 3.5–3.8 and 5.0 Å (Zr···Zr node distortions).^25^ New contributions at 3.3 Å (Zr–C_COO_ distances indicating PFCA replacement of the ^–^OH/H_2_O pairs) and 4.1–4.8 Å (Zr···C_adjacent_ and Zr···F interatomic distances) provide additional evidence of PFCA binding. Collectively, these data confirm chemisorption at the Zr nodes as a key step in the overall capture mechanism and demonstrate the power of synchrotron PDF characterization to reveal critical adsorbent–PFAS interactions.

**Figure 4:**
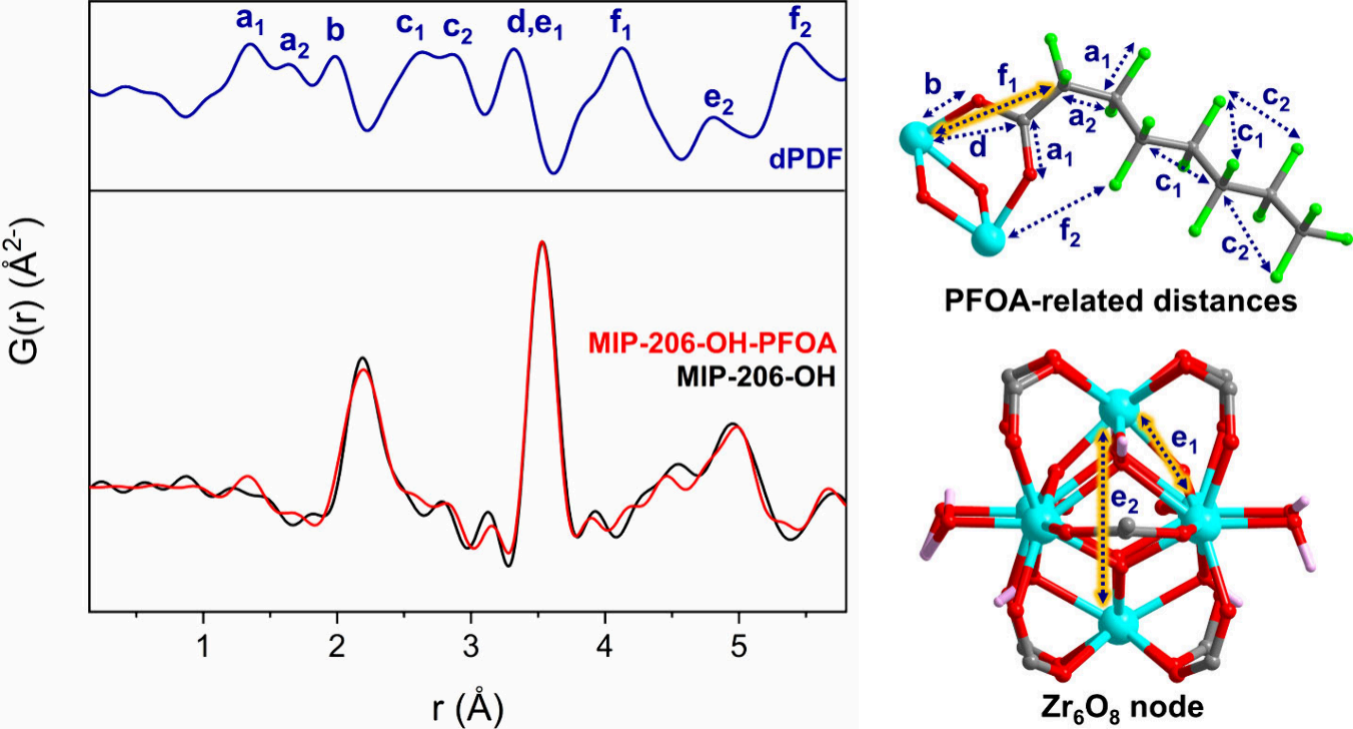
PDF data of MIP-206-OH before and after PFOA treatment. The corresponding dPDF signals (blue) are correlated to local distances within a Zr_6_O_8_ node loaded with PFOA (model constructed *in silico*).

In summary, the mesoporous MOFs MIP-206 and MIP-206-OH have been identified as highly effective materials for the removal of PFCA pollutants from water. Beyond mesopore filling, unsaturated Zr sites promote strong coordination binding, leading to exceptional performance for long-chain PFCAs, including ultrafast adsorption kinetics and high removal efficiencies across a range of concentrations. Detailed mechanistic studies including advanced synchrotron analyses provided comprehensive, atomic-level insight into these interactions. Pore functionalization with −OH groups was found to modulate adsorption performance, with effects strongly dependent on pollutant concentration. Overall, this work presents suitable sorbent candidates for PFAS decontamination and underscores the importance of multiple chemical interactions in enhancing adsorption. The findings provide a foundation for the rational design of next-generation MOF sorbents with a broad applicability for this pressing environmental challenge.

## Supplementary Material



## Notes

Synthesis and characterization of pristine MOFs, PFAS capture experiments and analysis, characterization of MOFs post-PFCA sorption, XAS and PDF analysis (PDF).
