# Supplementary material for: Ultrafast Capture of Per- and Polyfluoroalkyl Substances from Water by Mesoporous Zirconium Metal–Organic Frameworks
Source: ACS Mater Lett. 2026 Apr 24;8(6):1717–23. doi: 10.1021/acsmaterialslett.6c00186 (PMC13231454; doi:10.1021/acsmaterialslett.6c00186)
Supplement: Supplementary file 1 [file tz6c00186_si_001.pdf]

Ultrafast capture of per- and polyfluoroalkyl substances from water by mesoporous zirconium metal-organic frameworks

Sergio Marugán-Benito,[a,d] Rodrigo Gil-San-Millan,[a] María Alarcón del Río,[a] Lutz Ahrens,[b] Edward Loukopoulos*[a,c,d] and Ana E. Platero-Prats*[a,d]

[a] Departamento de Química Inorgánica, Facultad de Ciencias, Universidad Autónoma de Madrid, Campus de Cantoblanco, 28049 Madrid, Spain.

[b] Department of Aquatic Sciences and Assessment, Swedish University of Agricultural Sciences (SLU), Uppsala SE-75007, Sweden.

[c] Condensed Matter Physics Center (IFIMAC), Universidad Autónoma de Madrid, 28049, Campus de Cantoblanco, 28049 Madrid, Spain.

[d] Instituto de Catálisis y Petroleoquímica (ICP-CSIC), c/ Marie Curie 2, Madrid 28049, Spain.

SUPPORTING INFORMATION

**Table of contents**	Page
S1. Supplementary Methods	S2
S2. Synthesis and characterization of pristine MOFs	S3
S3. PFAS capture experiments and analysis	S13
S4. Characterization of MOFs post-PFCA sorption	S23
S5. X-Ray Absorption Spectroscopy analysis	S29
S6. X-Ray Pair Distribution Function analysis	S31
S7. Supplementary References	S33

S1. Supplementary Methods

Materials. All reagents were used as received from commercial suppliers without further purification. DUT-67 was prepared and characterized in accordance to a recent report by our group.[1]

NMR spectroscopy: ^{1}H and ^{19}F-NMR spectra were acquired on a Bruker Avance NEO-500 spectrometer, running at 500MHz. MOF samples were prepared by digesting a small portion (~5 mg) of each solid in a DMSO-d_6 (500 μL) / D_2O (100 μL) / HF (1 drop) or a KOH/ D_2O solution. To determine the amount of PFCA molecules binding to the MOF after capture, 2-fluorobenzoic acid was added as internal standard. Chemical shifts (δ) are reported in parts per million (ppm) relative to the residual solvent signal with a value of 2.50 ppm for DMSO-d_6.

Powder X-Ray Diffraction (PXRD): PXRD patterns were collected using a Bruker D8 diffractometer equipped with a copper source operating at 1600 W. The samples were ground and placed onto a borosilicate sample holder, and the surface was levelled with a clean microscope slide. The diffraction patterns were collected in continuous mode over a 2θ range of 2.5 to 45.0 degrees, with a step size of 0.02° and exposure times of 0.10 or 0.50 s per step. Calculated PXRD patterns from the corresponding single-crystal data were obtained using Mercury 3.8.[2]

Scanning Electron Microscope (SEM): SEM images were recorded on a FEI Nova NanoSEM 230 microscope.

Fourier-transform infrared (FT-IR) spectroscopy: Spectra were recorded on a PerkinElmer 100 spectrophotometer using a PIKE Technologies MIRacle Single Reflection Horizontal ATR Accessory from 4000–450 cm^{-1}.

Gas sorption studies: Low pressure N_2 sorption measurements were carried out at 77 K using a Micromeritics 3Flex Surface and Catalyst Characterization Analyzer system. Prior to analysis, the samples were activated under dynamic vacuum at 140 °C for 16 hours to remove all solvent molecules from the pores. Activation was performed on the degasser port of the instrument using a specific amount of MOF inside a 12 mm sample cell tube. The cell was then transferred to the analysis port of the instrument. After the measurement, the sample was re-weighed to obtain its

precise mass and perform data analysis. Pore-size-distribution (PSD) curves were obtained from the adsorption branches using non-local density functional theory (NLDFT) method for a cylinder pore in oxide surface.

Thermogravimetric Analysis (TGA): Measurements were carried out under air atmosphere using a TA Instruments Model TA-STD-Q600 analyzer. The temperature range applied for the analyses was from 40 °C to 800 °C, with a controlled heating rate of 10 $°C \cdot min^{-1}$.

S2. Synthesis and characterization of pristine MOFs

Synthesis of MIP-206: Isophthalic acid (1.10 g, 6.6 mmol) was weighed into a 25 ml Teflon reactor containing 5 ml of formic acid. The mixture was sonicated at room temperature for 5 min to form a homogeneous suspension. $ZrCl_4$ (2.00 g, 8.6 mmol) was then added to the suspension and the mixture was sonicated for another 10 minutes. The Teflon reactor was then sealed and placed in an autoclave, heated to 180 °C for 2 h and kept at that temperature for 20 h. A white microcrystalline solid was formed during this period. After allowing the mixture to cool to room temperature, the solid material was washed with water and acetone, then dried in the oven at 60ºC for 24 h. Yield: 1.90 g. ^{1}H-NMR in DMSO-d_6:D_2O:HF (Figure S8): δ 8.44 (t, J = 1.9 Hz, 1H), 8.14 (dd, J = 7.8, 1.8 Hz, 2H), 7.59 (t, J = 7.8 Hz, 1H). Other peaks: 2.05 (s, acetone).

Synthesis of MIP-206-OH: A similar synthetic protocol was employed, using instead 5-hydroxyisophthalic acid (0.72 g, 4.0 mmol) and $ZrOCl_2 \cdot 8\ H_2O$ (1.93 g, 6.0 mmol). After cooling, the resulting white solid was washed with water and acetone, then dried in the oven at 60ºC for 24 h. Yield: 1.4 g. ^{1}H-NMR in DMSO-d_6:D_2O:HF (Figure S9): δ 8.07 (s, 0.04H), 7.94 (t, J = 1.6 Hz, 1H), 7.53 (d, J = 1.6 Hz, 2H). Other peaks: 2.05 (s, acetone).

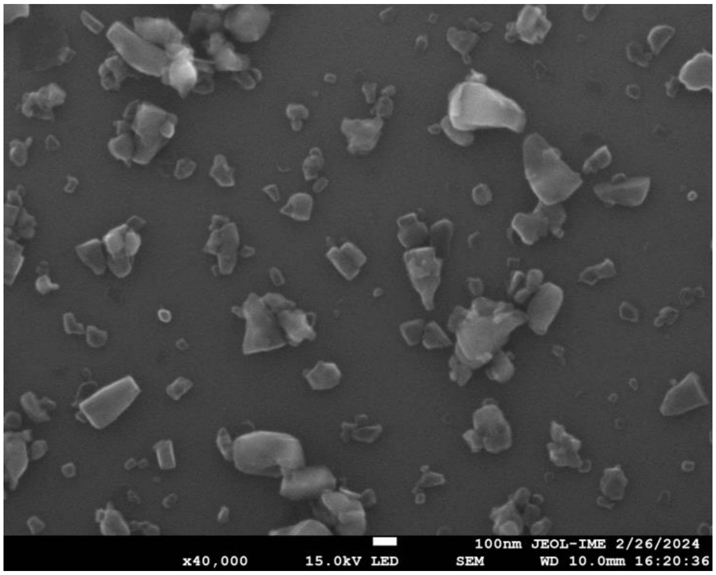

Figure S1. SEM image of the microcrystallites in as-made MIP-206.

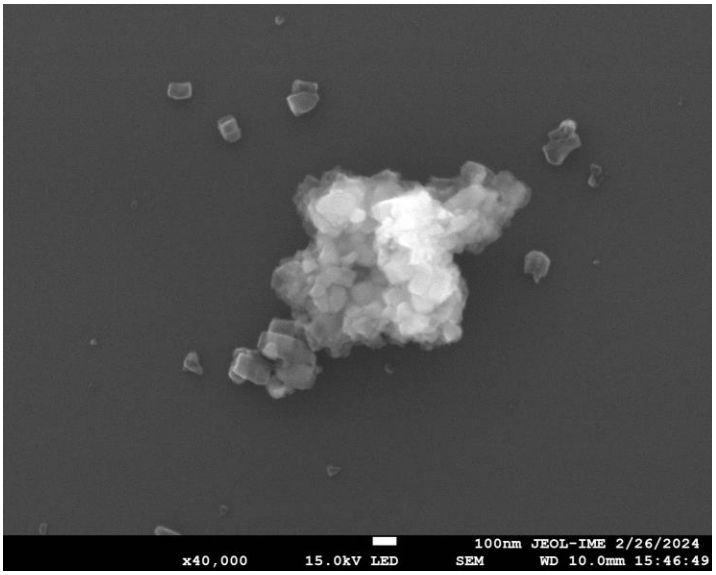

Figure S2. SEM image of the microcrystallites in as-made MIP-206-OH.

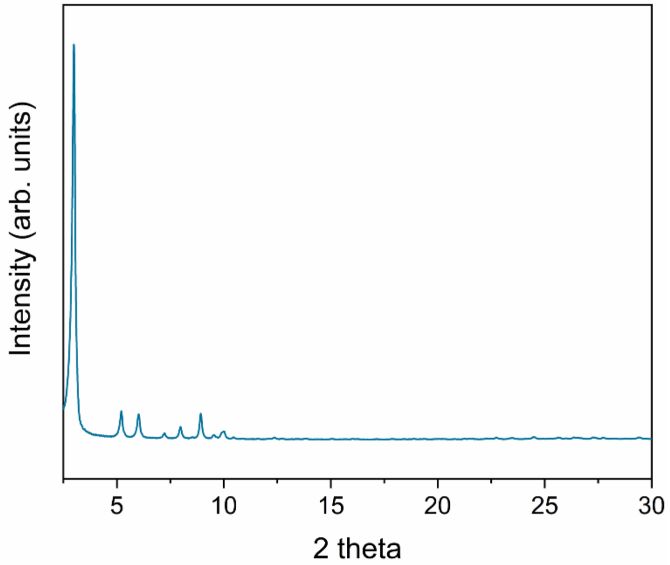

Figure S3. PXRD pattern of as-made MIP-206.

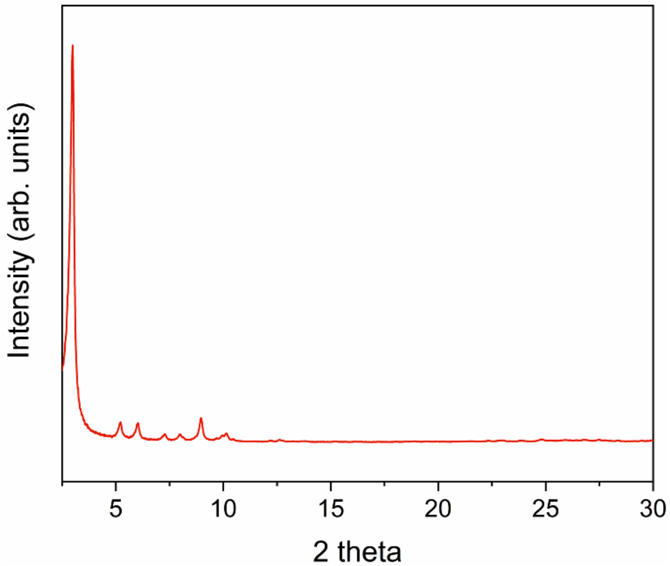

Figure S4. PXRD pattern of as-made MIP-206-OH.

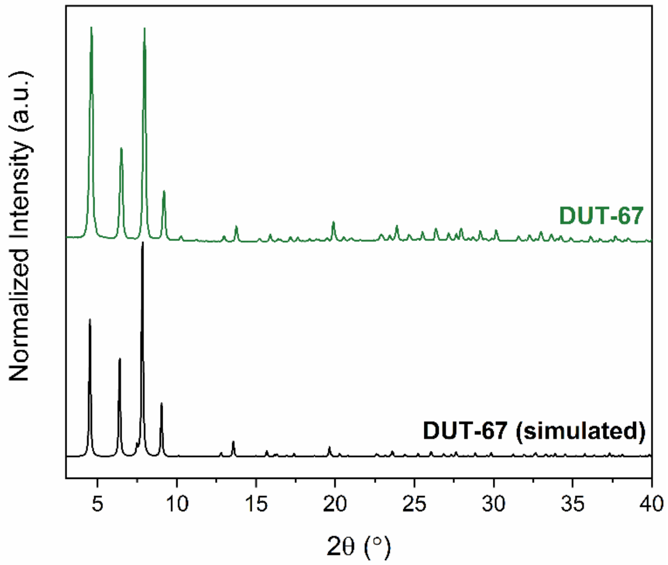

Figure S5. PXRD pattern of as-made DUT-67, compared to the one calculated from the reported crystal structure.

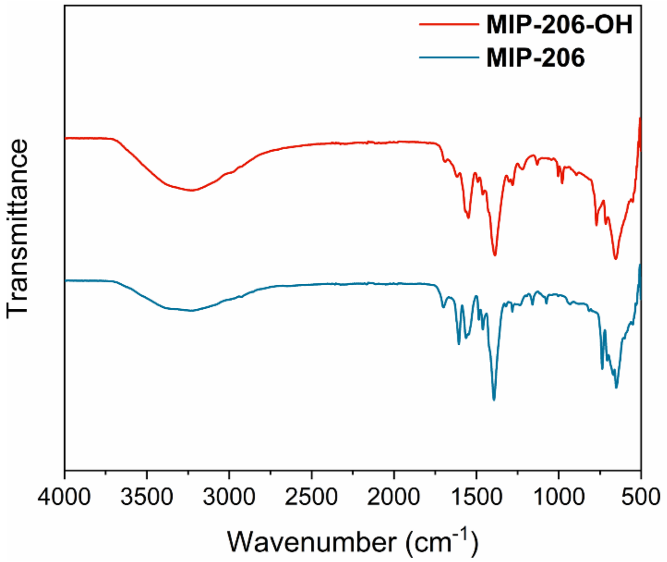

Figure S6. FT-IR spectra of as-made MIP-206 and MIP-206-OH. Characteristic peaks for both materials: 655 cm^{-1} (Zr-O bonds within the cluster nodes), 1395, 1455 and 1565 cm^{-1} (COO^- asymmetric and symmetric stretching), 1608 cm^{-1} (C=C bond stretching in the isophthalic moiety).

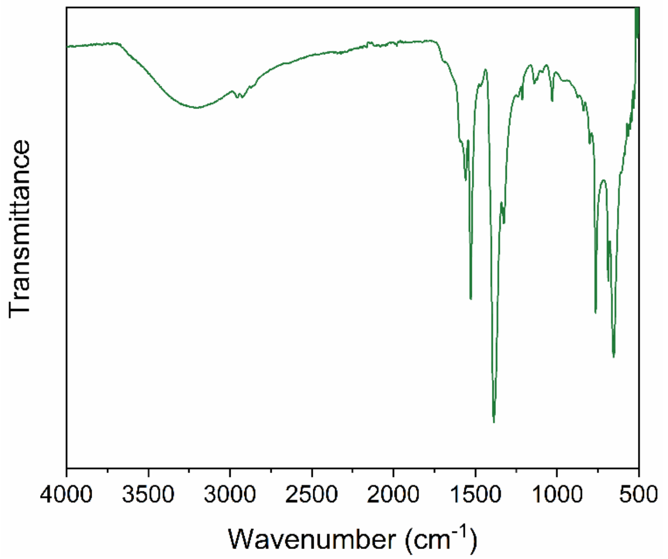

Figure S7. FT-IR spectra of as-made DUT-67. Characteristic peaks: 654 cm^{-1} (Zr-O bonds within the cluster nodes), 765 cm^{-1} (C-S bonds within the thiophene-based ligands), 1387 and 1529 cm^{-1} (COO^- asymmetric and symmetric stretching), 1570 cm^{-1} (C=C bond stretching within the thiophene ring).

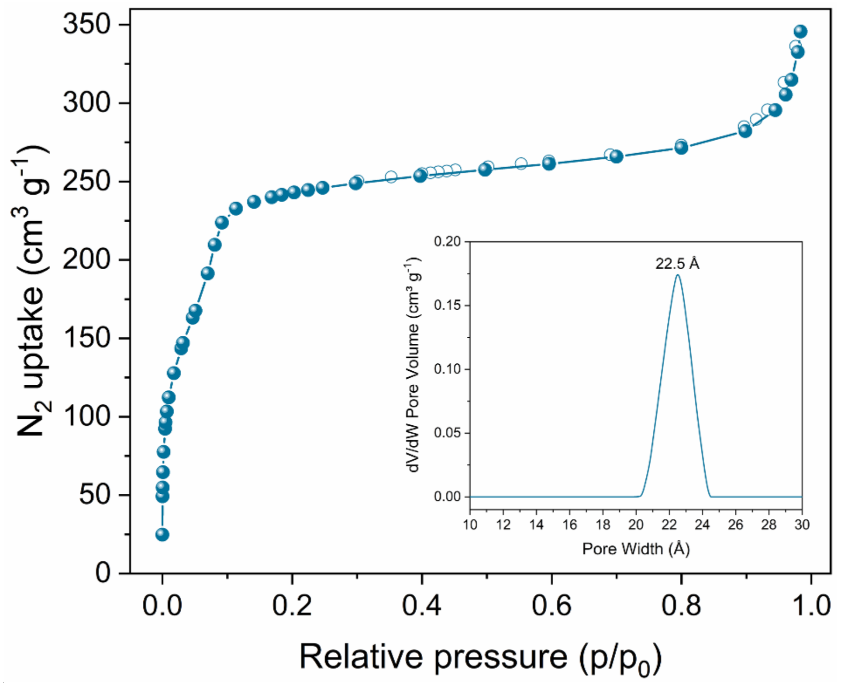

Figure S8. N_2 isotherm (linear scale) and corresponding pore size distribution analysis for MIP-206, recorded at 77 K.

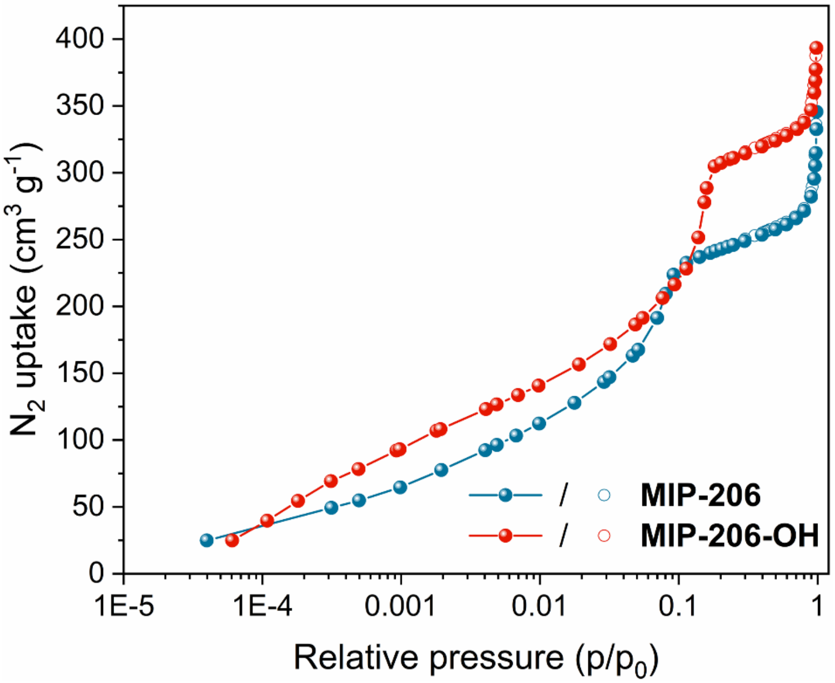

Figure S9. N_2 isotherm in semi-logarithmic scale for both materials of this study, recorded at 77 K.

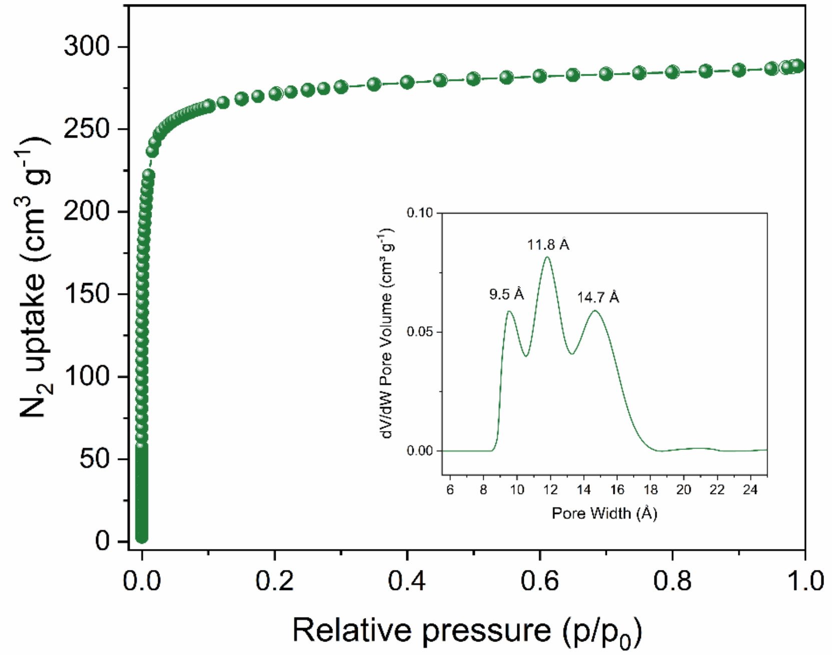

Figure S10. N_2 isotherm (linear scale) and corresponding pore size distribution analysis for DUT-67, recorded at 77 K.

Table S1. Nitrogen uptake, pore volume (calculated at $p/p_0 = 0.95$) and BET surface area values materials of the MOFs of this study, as calculated from the N_2 isotherms. The analogous reported values from the literature are also provided for comparison.

Material	Uptake ($cm^3\ g^{-1}$)	Pore volume ($cm^3\ g^{-1}$)	BET surface area ($m^2\ g^{-1}$)	Pore Size (nm)
MIP-206	295.4	0.45	1040±62	2.25
MIP-206 (reported)[3]	290	0.43	1024±41	2.38
MIP-206-OH	359.9	0.55	1230±56	2.55
MIP-206-OH (reported)[3]	350	0.54	1227±50	2.61
DUT-67	288.3	0.44	1099±4	0.95, 1.18, 1.47
DUT-67 (reported)[4]	287	0.44	1064	0.96, 1.17, 1.42

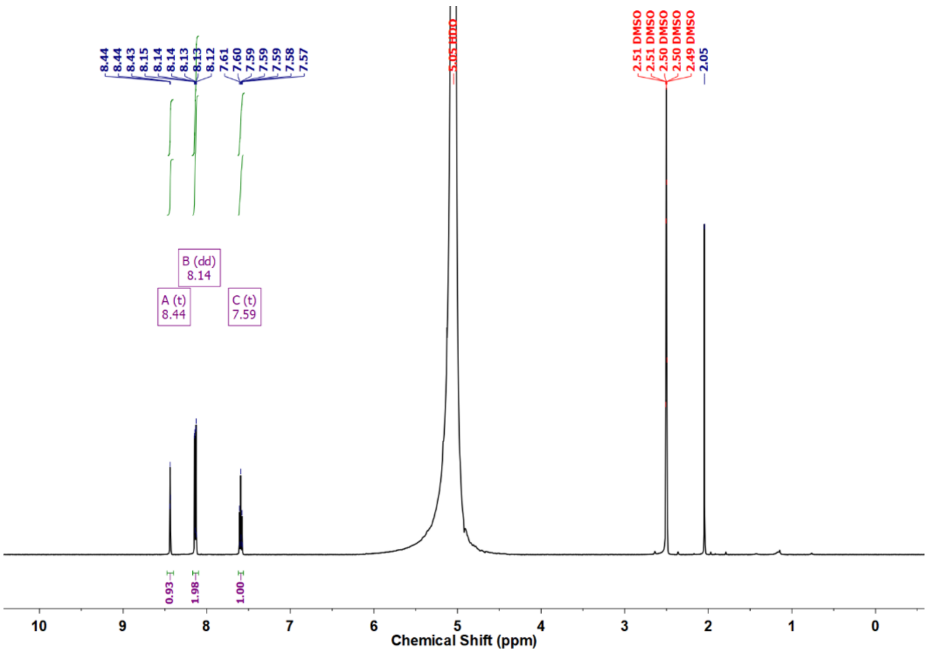

Figure S11. ^{1}H-NMR of as-made MIP-206 in DMSO-d_6:D_2O:HF (500 MHz).

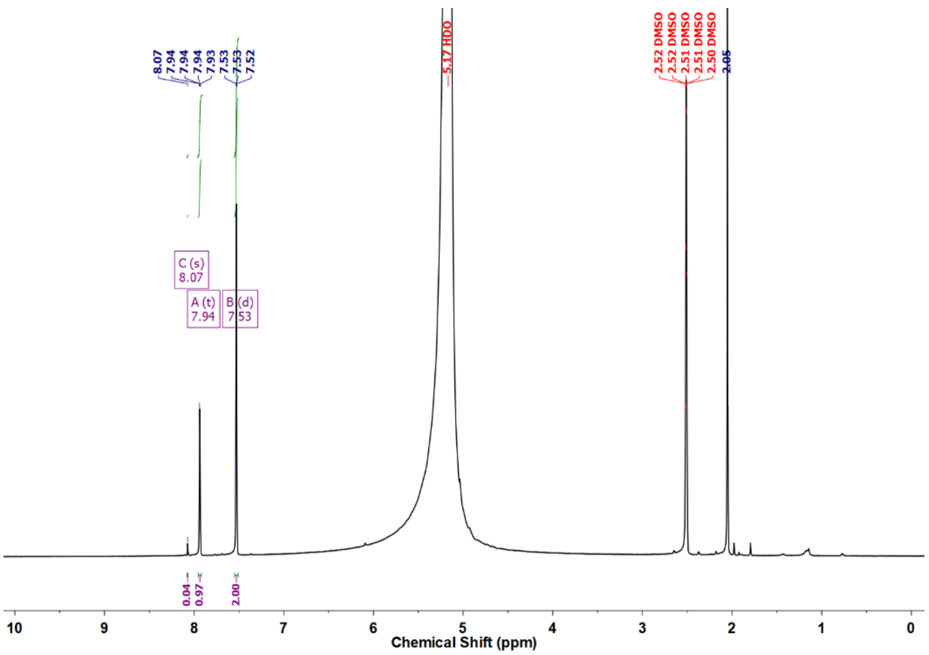

Figure S12. ^{1}H-NMR of as-made MIP-206-OH in DMSO-d_6:D_2O:HF (500 MHz).

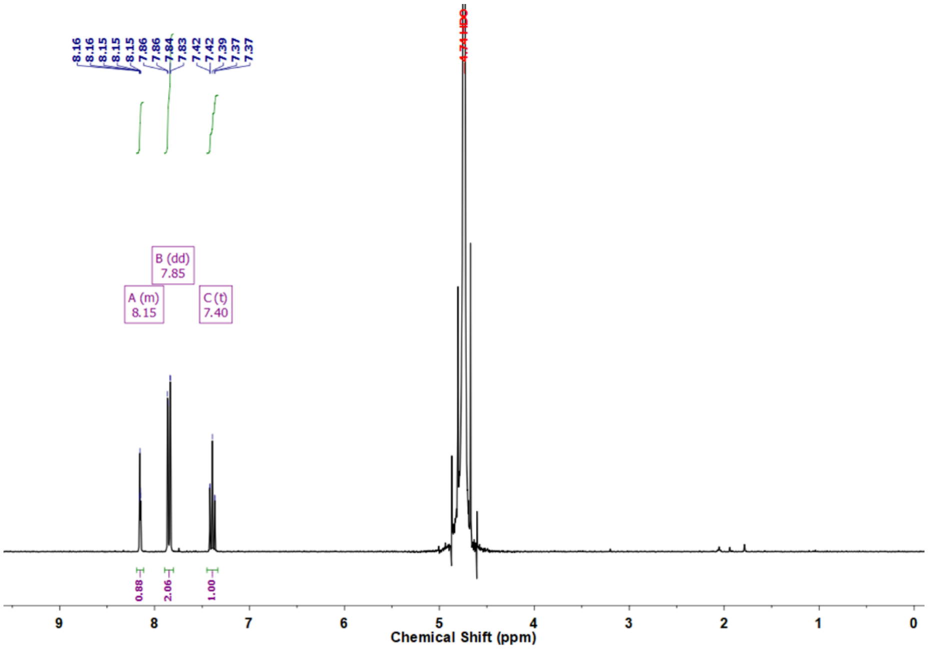

Figure S13. ^{1}H-NMR of as-made MIP-206 in KOH/D_2O. (500 MHz).

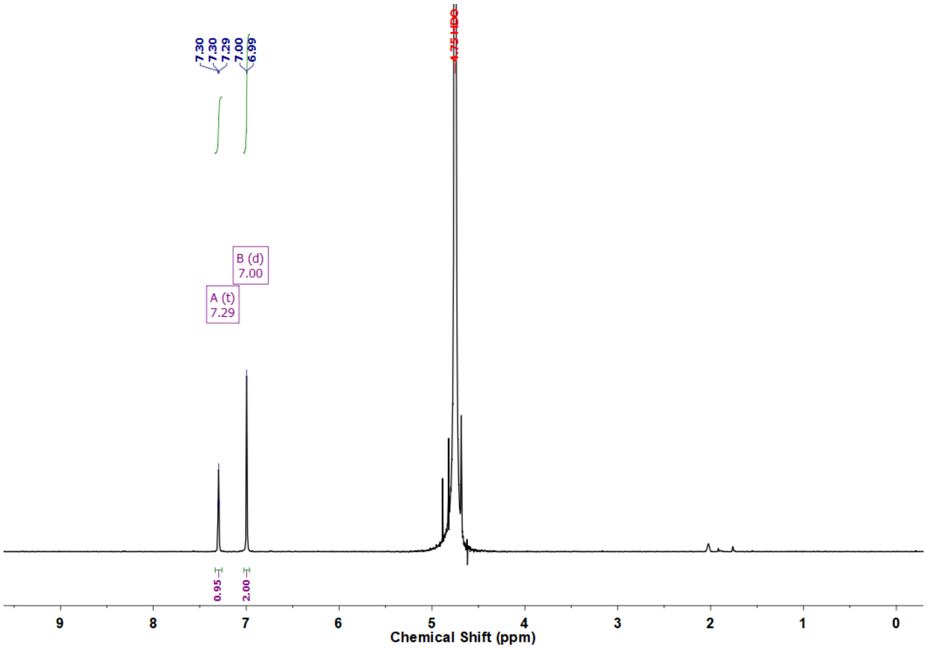

Figure S14. ^{1}H-NMR of as-made MIP-206-OH in KOH/D_2O. (500 MHz).

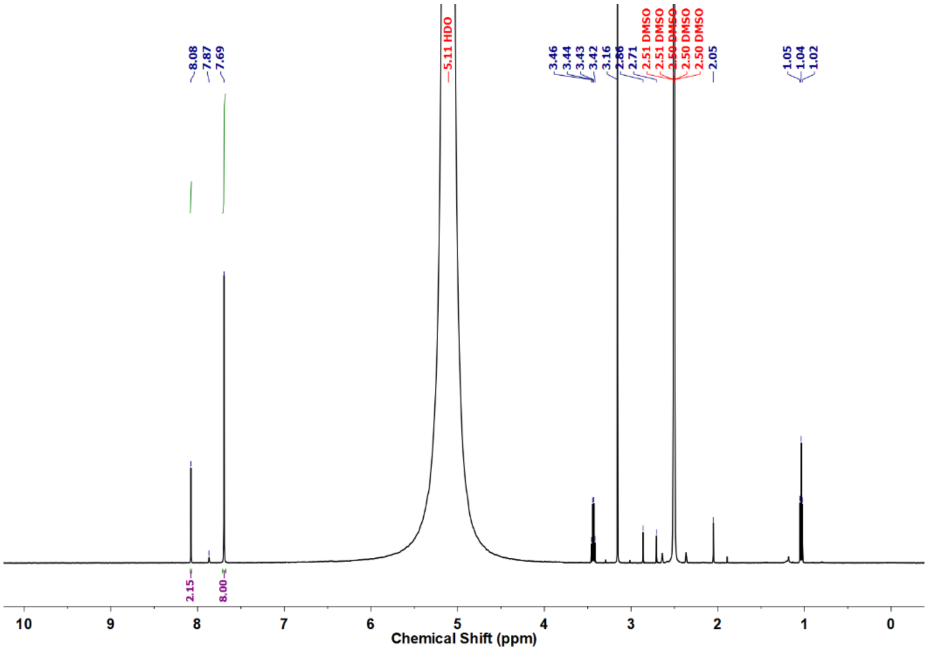

Figure S15. ^{1}H-NMR of as-made DUT-67 in DMSO-d_6:D_2O:HF (500 MHz).

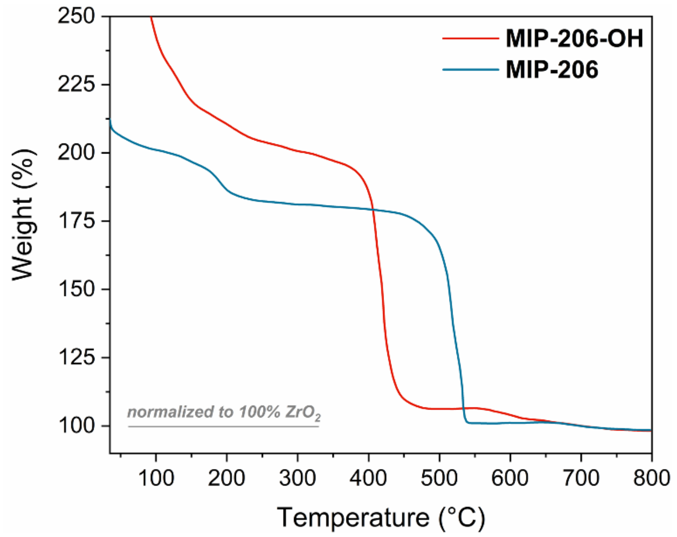

Figure S16. TGA graphs for as-made MIP-206 and MIP-206-OH (normalized to 100% ZrO_2 as the final residue). Initial weight loss up to 150 °C corresponds to solvent molecule removal. The main frameworks retain stability up to *ca.* stable up to 400 (MIP-206-OH) and 450 (MIP-206) °C, where decomposition of the organic linkers occurs, down to ZrO_2 by 600 °C.

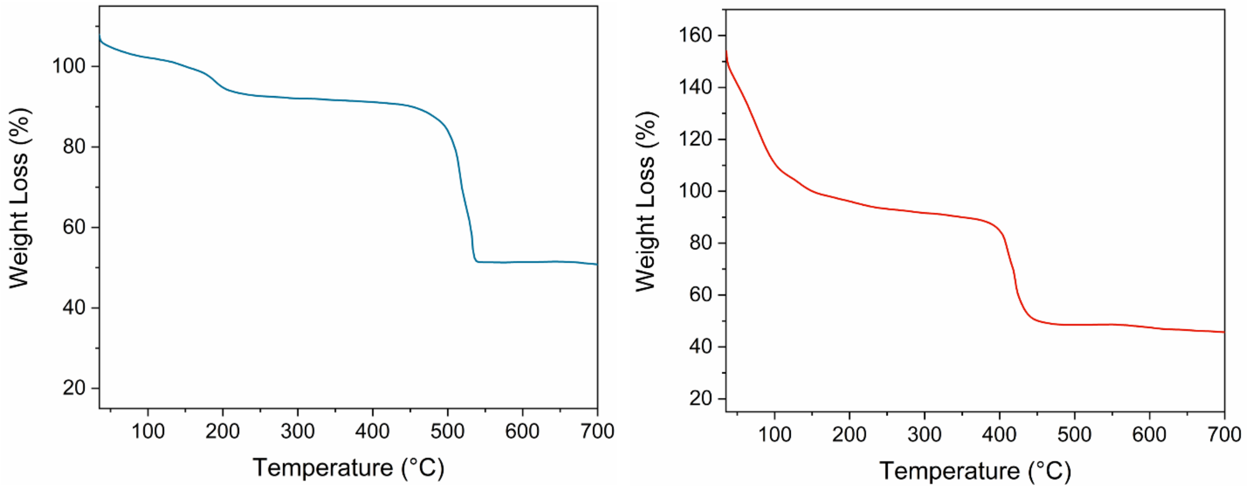

Figure S17. Individual TGA graphs of MIP-206 (left) and MIP-206-OH (right), considering a solvent-less framework with $^{-}OH/H_2O$ pairs instead of formate anions at 150 °C. For MIP-206: Estimated formula: (isophthalate)$_{18}$[Zr_{12}(μ_3-O)$_8$(μ_3-OH)$_8$(μ_2-OH)$_6$(OH)$_6$(H_2O)$_6$][Zr_6(μ_3-O)$_4$(μ_3-OH)$_4$(OH)$_4$(H_2O)$_4$]$_3$. Calcd. loss: 48.1%. Exper. loss: 48.6%. For MIP-206-OH: Estimated formula: (5-hydroxyisophthalate)$_{18}$[Zr_{12}(μ_3-O)$_8$(μ_3-OH)$_8$(μ_2-OH)$_6$(OH)$_6$(H_2O)$_6$][Zr_6(μ_3-O)$_4$(μ_3-OH)$_4$(OH)$_4$(H_2O)$_4$]$_3$. Calcd. loss: 50.1%. Exper. loss: 53.8%. Figure S12 shows very minor presence of formates within the structure, in agreement with the small deviation between values.

S3. PFAS capture experiments and analysis

General method for experiments in ppm pollutant concentrations: All capture tests were carried out by adding a predetermined amount of MOF adsorbent into a 20 mL capped vial containing an aqueous solution of an individual PFCA (either TFA, PFBA, PFHxA or PFOA) of already known concentration, in a dosage of 1.0 mg mL^{-1}. The mixture was stirred at 25 ºC and the capture was studied at different PFAS initial concentrations. All experiments were performed at least twice to ensure reproducibility. After the end of the designated time, the supernatant was then separated via centrifugation (14000 rpm for 2 minutes).

To evaluate the amount of fluorinated pollutant remaining in the supernatant, quantitative ^{19}F-NMR was performed using a trifluoroethanol (TFE)/D_2O internal standard of predetermined concentration. Each NMR spectra was collected using a tube containing 700 µL of the supernatant solution and 50 µL of the TFE/D_2O standard. The concentration of PFAS was related to the one of TFE using the following equation:

$$[PFAS] = \frac{I_{PFAS}}{I_{TFE}} \cdot [TFE] \quad (1)$$

In this equation [PFAS] and [TFE] are molar concentrations, while I_{PFAS} and I_{TFE} are the integrals of each compound from the ^{19}F-NMR spectrum. The $–CF_3$ peak of the pollutant (-80.7 ppm for PFOA, -75 ppm for TFA) was always used for these calculations. The following parameters were used to ensure quantitative integration, as already reported in the literature: R0 = 0 Hz, NS = 16 scans, D1 = 20 s, TE = 300 K.[5] Capture performance was then analysed by calculating the difference in PFAS concentration before (blank experiment) and after the reaction. After the capture experiment was complete and the supernatant was removed, the solid was decanted, washed with water and acetone (three times each), then dried at 60 ºC for 24 h. The material was then characterized as detailed in other Sections. Recycling studies for PFOA were performed for a total of 5 cycles; during each cycle the adsorbent was immersed to an aqueous PFOA solution of 100 ppm (MOF dosage of 1.0 mg mL^{-1}) for 10 min. The mixture was then centrifuged and the material was decanted. Removal efficiency was evaluated by analyzing the supernatant as previously described in the general method. Part of the isolated solid material was characterized with ^{19}F-NMR to confirm full desorption between cycles. The remaining solid was then used for a subsequent adsorption cycle. Additional PXRD characterizations were also performed to the solid samples after cycle 5, to further study the structural stability of the frameworks.

General method for experiments in ppb pollutant concentrations: Initially, a mixture of the target PFAS analytes (as found in Table S2) was made, with an initial concentration of 0.1 mg/mL for each individual PFAS. Individual PFAS standards in either salt or liquid forms were dissolved in methanol (MeOH) and then combined into a single PFAS stock solution, which was then diluted with MilliQ water to reach the desired concentration.

The capture tests were then carried out via the following process: 20 mg of MOF adsorbent were first added into a 50 mL falcon tube. 20 mL of MilliQ water solution were then added, already spiked with 0.1 mL of the solution containing the PFAS analytes mixture (final concentration for each individual PFAS: 0.25 μg/mL). The tube was shaken at 25 ºC for 24 hours. All experiments were performed at least twice to ensure reproducibility. After the end of the designated time, the supernatant was then separated via centrifugation (3000 rpm for 15 minutes). 0.25 mL of the supernatant were then transferred to a chromatographic glass vial containing 0.20 mL of methanol and 0.05 mL of the internal standard. ^{13}C mass-labelled internal standards for PFCAs were used throughout the analysis (Table S2).

The analysis of PFAS was performed as detailed elsewhere[6] using an ultra-high performance liquid chromatography with tandem mass spectrometry (UPLC-MS/MS) instrument, a Sciex TQ-S 3500 triple quadrupole mass spectrometer furnished with a heated electrospray ionization source, from Waters (Mildford, MA, U.S.). Chromatographic separation was performed in a Phenomenex Kinetix® C18 pre-column (1.7 μm, 100 A). For analysis, a Phenomenex Gemini® 3 μm C18 HPLC analytical column (2 x 2.1 mm, 3 μm, 110 A) with a Phenomenex KJ0-4282 analytical guard column was used. Separation was achieved with Milli-Q water 10 mM ammonium acetate (A) and pure MeOH (B), as mobile phases at a constant flow rate of 600 $\mu L\ min^{-1}$ and constant temperature of 40ºC in the column. The autosampler temperature was held constant at 15°C. The mobile phase gradient starting with the fraction of MeOH at 5% was programmed as: 0.0 min 5% B, 0.0-0.1 min 55% B, 0.1-4.4 min 99% B, 4.5-8-0 min 99% B,8.0 to 8.5 min 5% B, 8.5 to 12 min 5% B. The injection volume was 10 μL. Analytes were ionized under negative ionization mode (ESI). Fits of $R^2 > 0.99$ were obtained from the generated calibration curves.

Table S2. Determination parameters of PFCA target analytes in the UPLC-MS-MS instrument. Mass labelled internal standards were available for most individual analytes.

PFCA analyte	Formula	Precursor mass (Q1, m/z)	Product mass (Q3, m/z)	Retention time (min)	Declustering potential (V)	Collision energy (V)	Collision cell exit Potential (V)
PFPeA	$C_5HF_9O_2$	262.9	218.9	1.27	-5	-12	-11
PFHxA	$C_6HF_{11}O_2$	313.0	268.9	1.56	-35	-10	.14
PFHpA	$C_7HF_{13}O_2$	362.9	318.9	1.93	-40	-14	-11
			169.0			-24	-5
PFOA	$C_8HF_{15}O_2$	413.0	369.1	2.32	-45	-16	-15
			169.1			-24	-5
PFNA	$C_9HF_{17}O_2$	463.0	219.0	2.71	-50	-24	-9
			168.9			-26	-9
PFDA	$C_{10}HF_{19}O_2$	513.0	268.9	3.09	-55	-26	-11
			218.9		-50	-26	-9
PFUnDA	$C_{11}HF_{21}O_2$	563.0	268.9	3.43	-60	-18	-9
					-55	-26	-11
PFDoDA	$C_{12}HF_{23}O_2$	613.0	569.0	3.74	-65	-18	-11
			318.9			-28	-13

Internal standards used							
**Acronym**	**Molecular formula**	**Precursor mass (Q1, m/z)**	**Product mass (Q3, m/z)**	**Retention time (min)**	**Declustering potential (V)**	**Collision energy (V)**	**Collision cell exit Potential (V)**
13C4-PFBA	$^{13}C_4F_7O_2$	217.0	172.0	1.06	-25	-14	-9
13C5-PFPeA	$^{13}C_5F_9O_2$	268.0	223.0	1.27	30	-12	-9
13C5-PFHxA	$^{13}C_5{}^{12}C_1F_{11}O_2$	318.0	273.0	1.56	-35	-14	-13
13C4-PFHpA	$^{13}C_4{}^{12}C_3F_{12}O_2$	367.0	172.0	1.93	-40	-24	-7
13C8-PFOA	$^{13}C_8F_{15}O_2$	421.0	376.0	2.32	-45	-16	-17
13C9-PFNA	$^{13}C_9F^{17}O_2$	472.0	172.0	2.71	-50	-26	-9
13C6-PFDA	$^{13}C_6{}^{12}C_4F_{19}O_2$	519.0	474.0	3.09	-75	-14	-19
13C7-PFUnDA	$^{13}C_7{}^{12}C_4F_{21}O_2$	570.0	525.0	3.43	-90	-18	-9
13C3-PFDoDA	$^{13}C_2{}^{12}C_{10}F_{23}O_2$	615.0	570.0	3.74	-65	-18	-11

Table S3. Initial PFCA removal efficiency values at ppm scale for the materials of this study. Experimental conditions: 5 mg of MOF immersed to 5 mL of PFCA solutions for 24 h unless noted. All experiments were performed at least two times and average values are reported.

MOF material	PFCA tested	Concentration (ppm)	Removal Efficiency (%)
MIP-206	PFOA	50	>99
MIP-206	PFOA	100	>99
MIP-206	PFOA	500	93.1
MIP-206	PFOA	1000	27.0
MIP-206	PFHxA	100	>99
MIP-206	PFBA	100	46.5
MIP-206	PFBA	500	23.6
MIP-206	TFA	100	35.0
MIP-206-OH	PFOA	50	>99
MIP-206-OH	PFOA	100	>99
MIP-206-OH	PFOA	500	>99
MIP-206-OH	PFOA	1000	>99
MIP-206-OH	PFHxA	100	>99
MIP-206-OH	PFBA	100	46.3
MIP-206-OH	PFBA	500	36.6
MIP-206-OH	TFA	100	40.0
DUT-67	PFOA	50	>99
DUT-67	PFOA	100	>99
DUT-67	PFOA[a]	100[a]	44.7
DUT-67	PFOA	500	64.9
DUT-67	PFHxA	100	65.1
DUT-67	PFBA	100	29.6
DUT-67	TFA	100	29.4

[a] Equilibrium time: 30 min

Table S4. PFOA removal efficiency of MIP-206-OH over various amounts of time. Experimental conditions: 5 mg of MOF immersed to 5 mL of 100 ppm PFOA aqueous solution. All experiments were performed at least two times and average values are reported.

Time (min)	Removal Efficiency (%)
1440	100
480	100
240	100
120	100
60	100
30	100
20	100
15	100
10	100
5	100
2	100
1	100
0.5	100

Table S5. Reported equilibrium times of PFOA adsorption kinetics for the highest-performing MOF materials of the literature, including the one presented in this work.

MOF material	PFOA concentration (ppm)	Equilibrium time (t_e)	Removal efficiency at equilibrium (%)	Ref.
MIP-206-OH	100	0.5 min	100	This work
NU-1000	100	1 min	100	7
UiO-66	500	10 min	~65[a]	8
UiO-66-F4	500	10 min	~76[a]	8
UiO-67	500	< 60 min	100[a]	9
Cr-MIL-101-QDMEN	1000	< 60 min	N/A	10
Cr-MIL-101	1000	< 60 min	N/A	10
Cr-MIL-101-DMEN	1000	< 60 min	N/A	10
Cr-MIL-101-NMe_3	1000	< 60 min	N/A	10
Cr-MIL-101-NH_2	1000	< 60 min	N/A	10
UiO-66-NH-PAOQ	200	60 min	86.9	11
PCN-999	1000	12 h	100	12

[a] Calculated from the reported adsorption capacities.

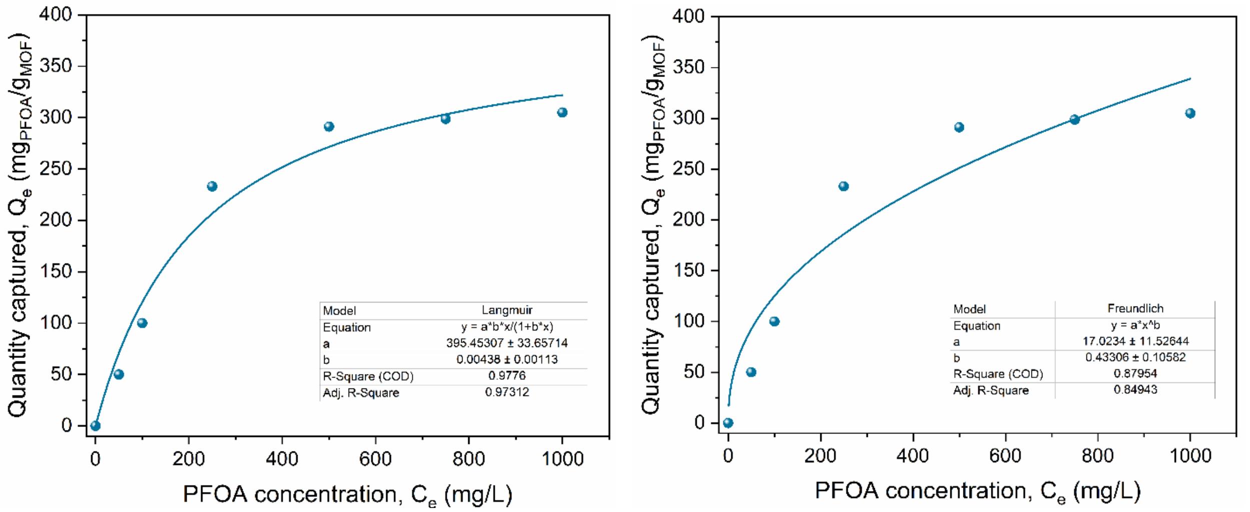

Figure S18. PFOA adsorption capacity studies using MIP-206, fitted with the Langmuir (left) or the Freundlich (right) model.

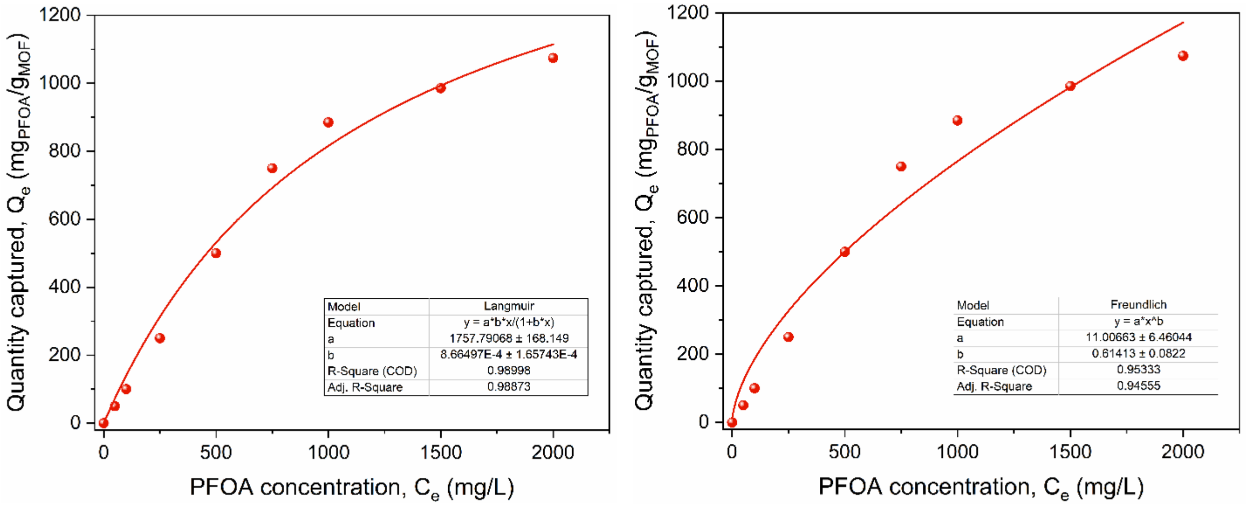

Figure S19. PFOA adsorption capacity studies using MIP-206-OH, fitted with the Langmuir (left) or the Freundlich (right) model.

Table S6. PFOA adsorption capacity data of this work, in comparison to the highest-performing MOF materials reported in the literature. All values refer to maximum adsorption capacities as determined by the Langmuir model.

MOF material	Maximum PFOA adsorption capacity[a] (mg/g) (ppm)	Concentration range (ppm)	Ref.
UiO-67-F2	3060	150-3232	[13]
TFA-MOF-808	2496	100-800	[14]
**MIP-206-OH**	1758	50-2000	This work
UiO-67	1589	500-3232	[13]
MOF-808	1581	100-800	[14]
UiO-66-$N(CH_3)_3^+$	1178	0-1000	[13, 15]
PCN-999	1089	100-5000	[12]
Cr-MIL-101-QDMEN	754	0-250	[10]
PCN-1002	632	100-2000	[16]
Fe-BTC	548	50-1000	[13, 17]
Cr-MIL-101-DMEN	534	0-250	[10, 15]
Cr-MIL-101-NMe_3	493	0-250	[10]
MIL-101-Fe	490	50-1000	[10, 17]
UiO-66-F4	467	0-350	[8]
Cr-MIL-101	460	0-250	[10]
MIL-100-Fe	427	50-1000	[17]
UiO-66-L_3	403	0-100	[8, 11]
**MIP-206**	395	50-2000	This work
UiO-66	388	0-600	[8, 17]
MIL-96-RHPAM2	340	0-1000	[18]
Cr-MIL-101-NH_2	290	0-250	[10]
Ce-BTC	210	N/A	[17]
PCN-1001	174	100-2000	[16]
Mn-BTC	130	N/A	[17]

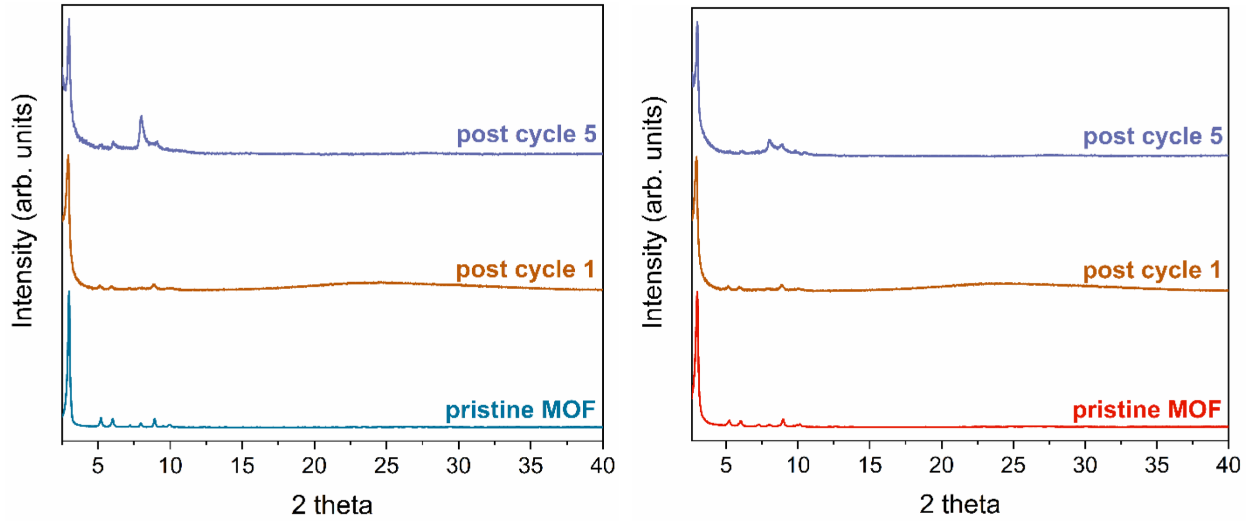

Figure S20. PXRD patterns of MIP-206 (left) and MIP-206-OH (right) materials, after different cycles of PFOA sorption (100 ppm).

Table S7. Actual concentrations of compounds used for the PFCA capture tests with competing ions.

Compound used	Concentration (ppm)
$Mg(NO_3)_2$	567
$CaSO_4$	567
NaCl	567
$FeCl_3$	567
$NaNO_3$	600
$Al(NO_3)_3$	433
Na_2SO_4	600

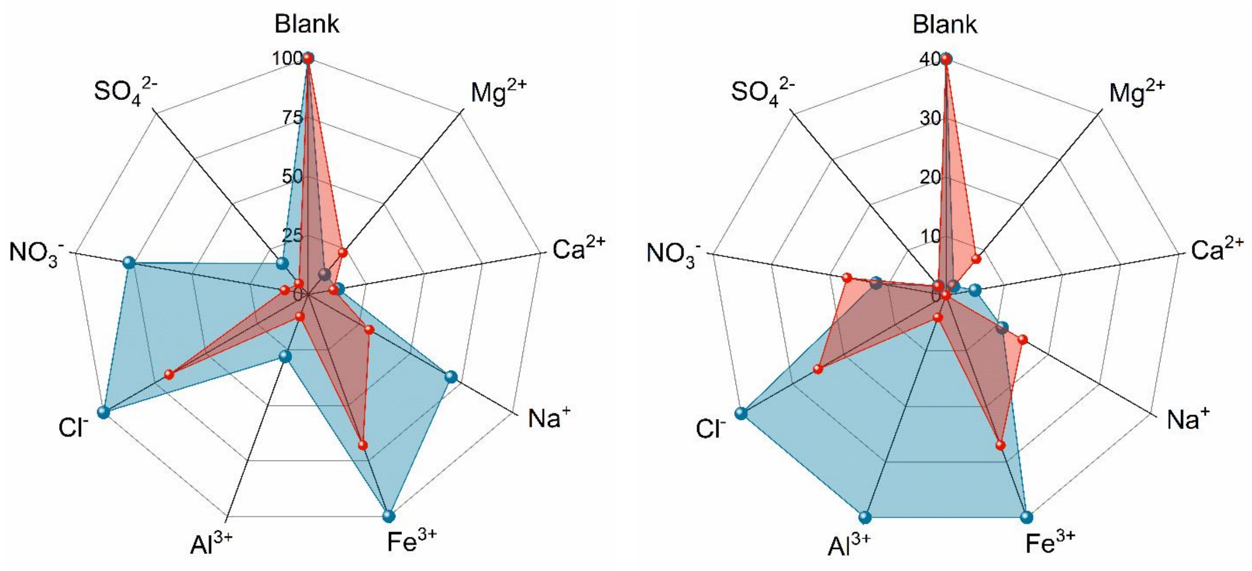

Figure S21. TFA (left) and PFHxA (right) removal efficiencies (C_0 = 100 ppm) of MIP-206 (blue) and MIP-206-OH (red) in the presence of various ionic species (2-10 mM).

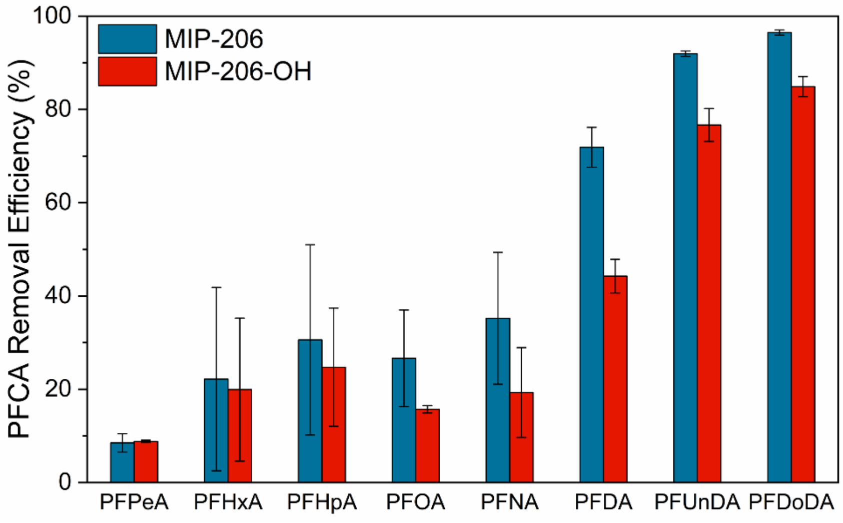

Figure S22. PFCA removal efficiencies of MIP-206 and MIP-206-OH at ppb levels ($[PFAS]_{total}$ = 250 ppb). All experiments were performed at least two times and average values are reported.

S4. Characterization of MOFs post-PFCA sorption

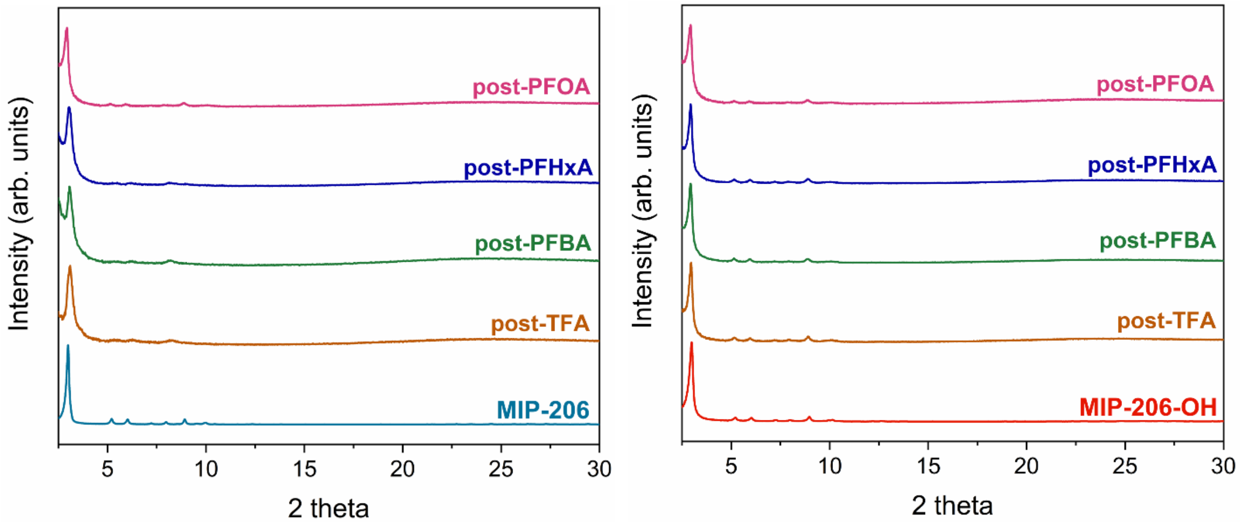

Figure S23. PXRD patterns of MIP-206 (left) and MIP-206-OH (right) materials, after PFCA sorption (100 ppm) and subsequent washes with acetone and water.

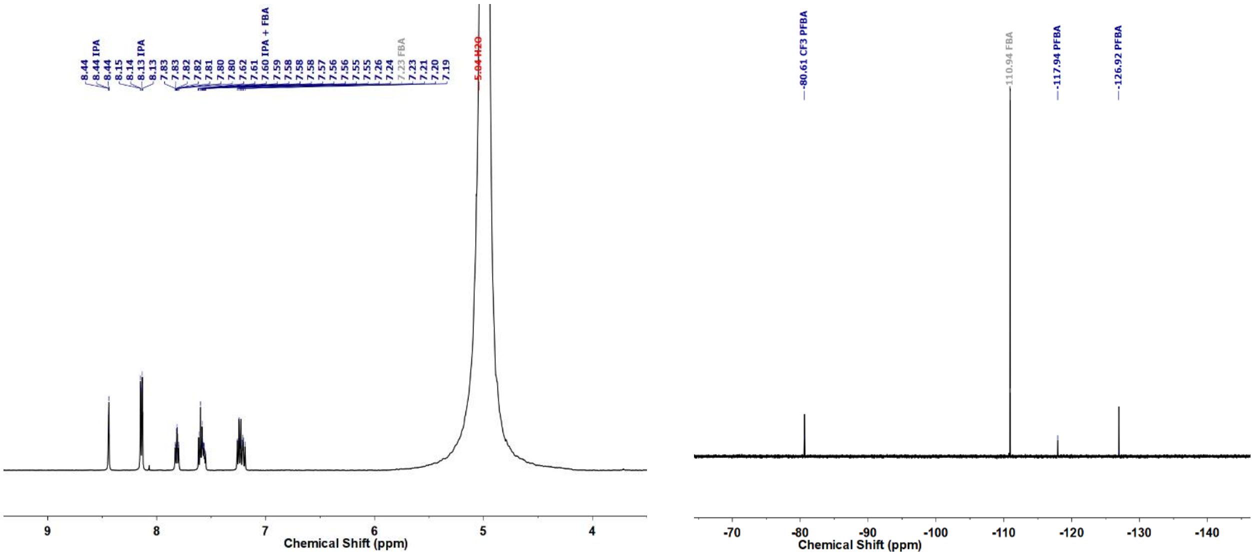

Figure S24. Representative ^{1}H-(left) and ^{19}F-(right) NMR of MIP-206 post-PFBA capture and after multiple washes with water and acetone, revealing that PFBA molecules remain strongly bound. Peaks of the internal standard 2-fluorobenzoic acid are also labelled. Quantification of these results and all PFCA-loaded solids is detailed in Tables S8-S9.

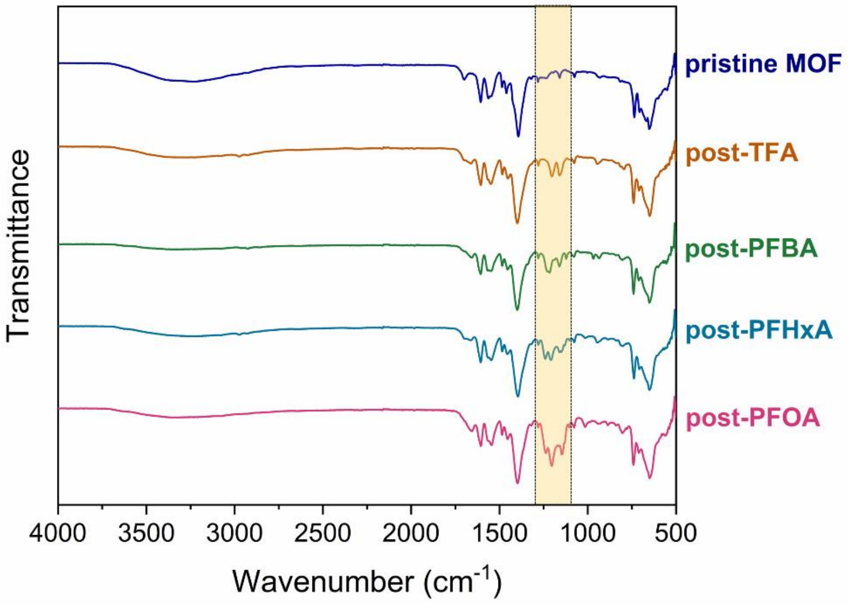

Figure S25. FT-IR spectra of MIP-206 after PFCA sorption. The additional peaks in the C-F bond region are highlighted.

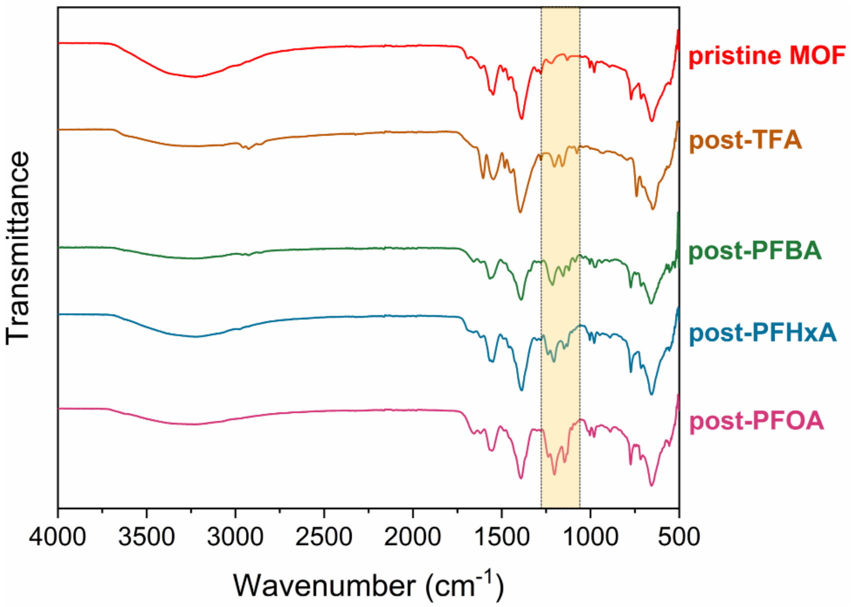

Figure S26. FT-IR spectra of MIP-206-OH after PFCA sorption. The additional peaks in the C-F bond region are highlighted.

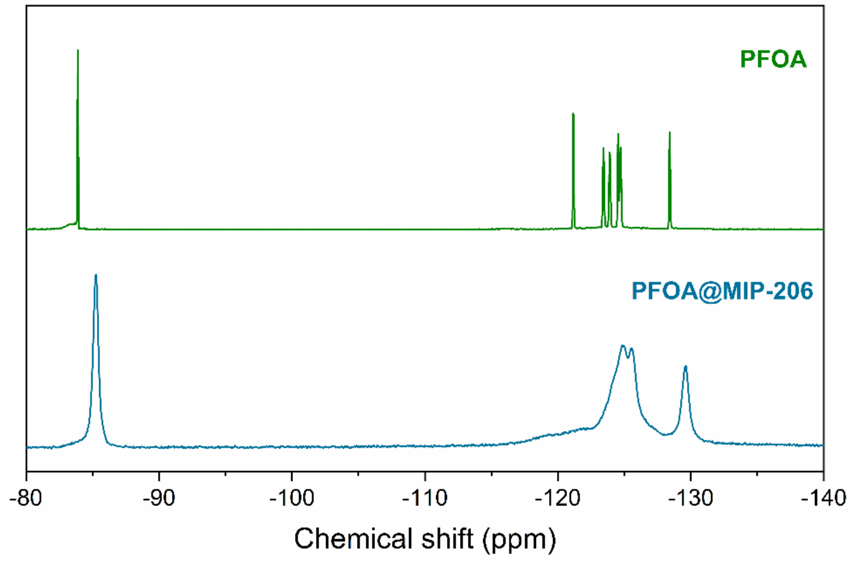

Figure S27. Solid-state ^{19}F-NMR spectra of PFOA and MIP-206 after PFCA sorption.

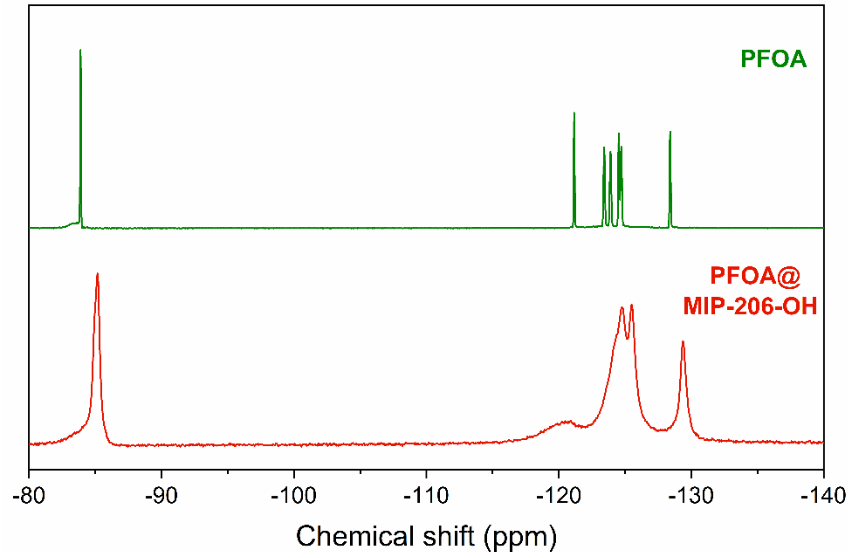

Figure S28. Solid-state ^{19}F-NMR spectra of PFOA and MIP-206-OH after PFCA sorption.

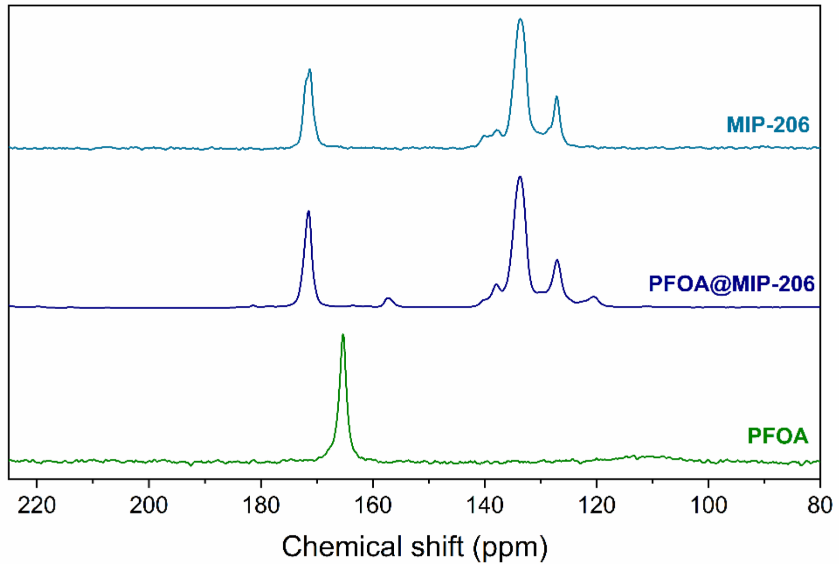

Figure S29. Solid-state ^{13}C-NMR spectra of PFOA, MIP-206 and MIP-206 after PFCA sorption.

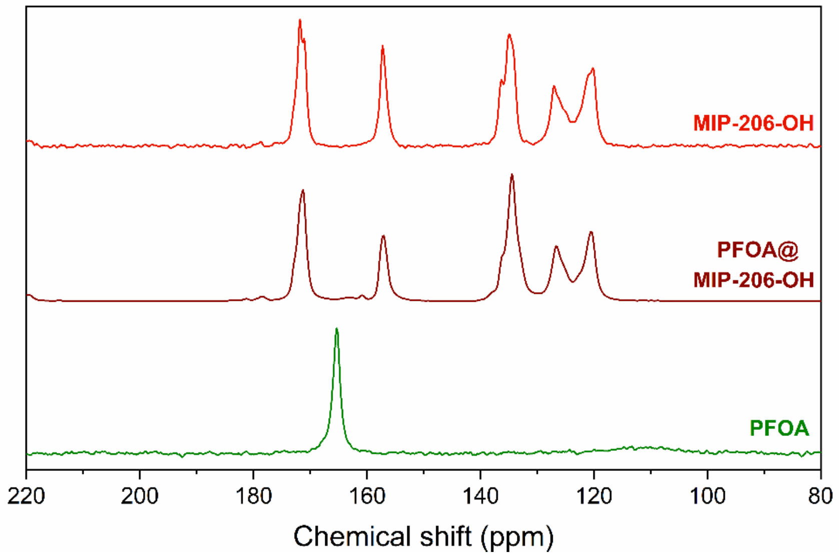

Figure S30. Solid-state ^{13}C-NMR spectra of PFOA, MIP-206-OH and MIP-206-OH after PFCA sorption.

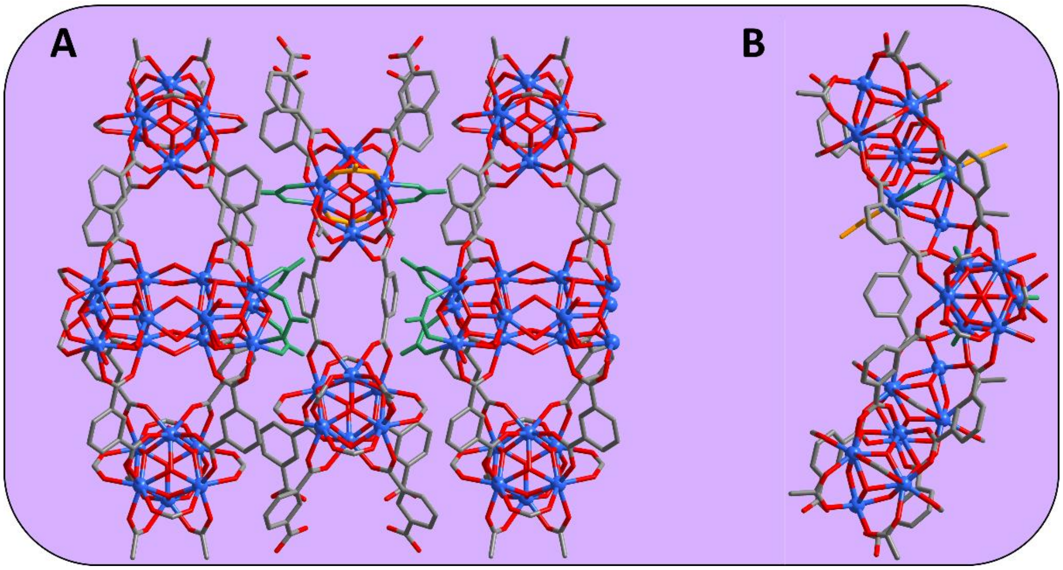

Figure S31. Structural views of MIP-206, showing the directionality of potential PFCA binding locations within the inorganic clusters. Positions that stereochemically prohibit binding are marked in green, and positions that allow binding are marked in orange. **(A)** View of the 2D layers shows that all 6 potential binding positions per $Zr_{12}O_{22}$ node and 2 per Zr_6O_8 node point towards the same layers in close proximity and cannot accommodate PFCAs. **(B)** View of the same segment along the *c* axis shows that 2 positions per Zr_6O_8 node point towards the mesopore and therefore represent potential PFCA locations after capture.

Table S8. Quantification of coordinated PFCA molecules in MIP-206 and MIP-206-OH after various capture conditions. All experiments were performed at least two times and average values are reported.

**PFCA type**	**Pollutant concentration (ppm)**	**PFCA molecules per Zr_6O_8 node**	
		**MIP-206**	**MIP-206-OH**
PFOA	100	0.38	0.39
PFHxA	100	0.37	0.22
PFBA	100	0.27	0.15
PFBA	500	0.72	0.60
TFA	100	0.32	0.23

Table S9. Detailed presentation of the PFCA adsorption performance of MIP-206 and MIP-206-OH using various metrics. Note: this Table only contains entries for which the post-capture solid was characterized to quantify the PFCA amount (i.e., entries detailed in Table S8).

MOF material	PFCA tested	Amount of MOF used		Amount of Initial PFCA				Initial ratio	Removal Efficiency (%)	Removal capacity		PFCA amount captured in MOF via coordination		% of occupied Zr_6O_8 metal sites	PFCA amount retained in MOF pore
		(mg)	(mmol)	(mg)	(mmol)	(ppm)	(mmol /L)	($mmol_{PFCA}$ per $mmol_{MOF}$)		(mg_{PFCA} / g_{MOF})	($mmol_{PFCA}$ / $mmol_{MOF}$)	($mmol_{PFCA}$ / Zr_6 node of MOF)	($mmol_{PFCA}$ / $mmol_{MOF}$)		($mmol_{PFCA}$ / $mmol_{MOF}$)
MIP-206	PFOA	5	0.0007	0.50	0.0012	100	0.24	1.7	100%	100	1.7	0.34	1.7	28.33%	0.0
MIP-206	PFHxA	5	0.0007	0.50	0.0016	100	0.32	2.3	100%	100	2.3	0.37	1.85	30.83%	0.5
MIP-206	PFBA	5	0.0007	0.50	0.0023	100	0.47	3.4	47%	47	1.6	0.27	1.35	22.50%	0.2
MIP-206	PFBA	5	0.0007	2.50	0.0117	500	2.34	16.9	24%	118	4.0	0.72	3.6	60.00%	0.4
MIP-206	TFA	5	0.0007	0.50	0.0044	100	0.88	6.4	35%	35	2.2	0.32	1.6	26.67%	0.6
MIP-206-OH	PFOA	5	0.0007	0.50	0.0012	100	0.24	1.8	100%	100	1.8	0.39	1.95	30.00%	0.0
MIP-206-OH	PFHxA	5	0.0007	0.50	0.0016	100	0.32	2.4	100%	100	2.4	0.22	1.1	18.33%	1.3
MIP-206-OH	PFBA	5	0.0007	0.50	0.0023	100	0.47	3.5	46%	46	1.6	0.15	0.75	12.50%	0.9
MIP-206-OH	PFBA	5	0.0007	2.50	0.0117	500	2.34	17.6	37%	183	6.4	0.60	3.00	50.00%	3.4
MIP-206-OH	TFA	5	0.0007	0.50	0.0044	100	0.88	6.6	40%	40	2.6	0.23	1.15	19.17%	1.5

Table S10. Detailed presentation of the PFCA adsorption performance of MIP-206 and MIP-206-OH using various metrics, including entries where only the solution was analysed (i.e., entries not presented in Tables S8-S9).

MOF material	PFCA tested	Amount of MOF used		Amount of Initial PFCA				Initial ratio	Removal Efficiency (%)	Removal capacity	
		(mg)	(mmol)	(mg)	(mmol)	(ppm)	(mmol/L)	($mmol_{PFCA}$ per $mmol_{MOF}$)		(mg_{PFCA} / g_{MOF})	($mmol_{PFCA}$ / $mmol_{MOF}$)
MIP-206	PFOA	5	0.0007	0.25	0.0006	50	0.12	0.9	100%	50	0.9
MIP-206	PFOA	5	0.0007	1.25	0.0030	250	0.60	4.3	93%	233	4.0
MIP-206	PFOA	5	0.0007	2.50	0.0060	500	1.21	8.7	94%	469	8.2
MIP-206	PFOA	5	0.0007	3.75	0.0091	750	1.81	13.1	40%	300	5.2
MIP-206	PFOA	5	0.0007	5.00	0.0121	1000	2.42	17.5	27%	270	4.7
MIP-206-OH	PFOA	5	0.0007	0.25	0.0006	50	0.12	0.9	100%	50	0.9
MIP-206-OH	PFOA	5	0.0007	1.25	0.0030	250	0.60	4.5	100%	250	4.5
MIP-206-OH	PFOA	5	0.0007	2.50	0.0060	500	1.21	9.1	100%	500	9.1
MIP-206-OH	PFOA	5	0.0007	3.75	0.0091	750	1.81	13.6	100%	750	13.6
MIP-206-OH	PFOA	5	0.0007	0.50	0.0044	100	0.88	6.6	40%	40	2.6

Table S11. Evaluation of various regeneration protocols for MIP-206-OH to remove strongly bound PFOA molecules from the material.

Washing Solution	Concentration (M)	Temperature (ºC)	Time (hours)	PFOA molecules remaining after treatment (%)
HCl/MeOH	0.15	50	16	0
aq. Na_2SO_4	0.15	50	16	0
aq. NaCl	0.15	50	16	0

S5. X-Ray Absorption Spectroscopy analysis

Ex situ XAS data were collected at NOTOS beamline of the ALBA Synchrotron Light Source facility (Cerdanyola del Vallès, Barcelona, Spain, proposals 2024028145 and 2024028149). Samples (MOFs or reference systems) were finely ground and mixed with cellulose to prepare suitable pellets for data collection. Sample/cellulose ratios were estimated for each system considering the targeted absorption edge and the Zr content in each solid (as previously determined by chemical analyses). Zr foil and all samples were measured under room temperature and in transmission mode using Oken ionization chambers, filled with the appropriate mixture of gases. Energy scale have been calibrated at Zr foil edge (17998 eV). The raw spectra have been processed according to standard methods, using the Demeter XAS suite.[19] They have been normalized by subtracting pre-edge and post edge line fitted as low-order polynomial curves. The corresponding EXAFS signal has been then extracted in the range 3-12 $Å^{-1}$, k-squared weighted, and Fourier transformed (FT).

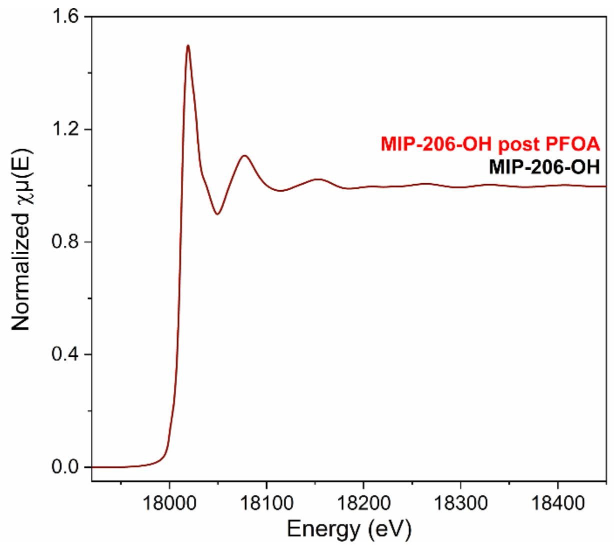

Figure S32. *Ex situ* XANES spectra comparison (Zr *K*-edge) of MIP-206-OH before and after capture of PFOA.

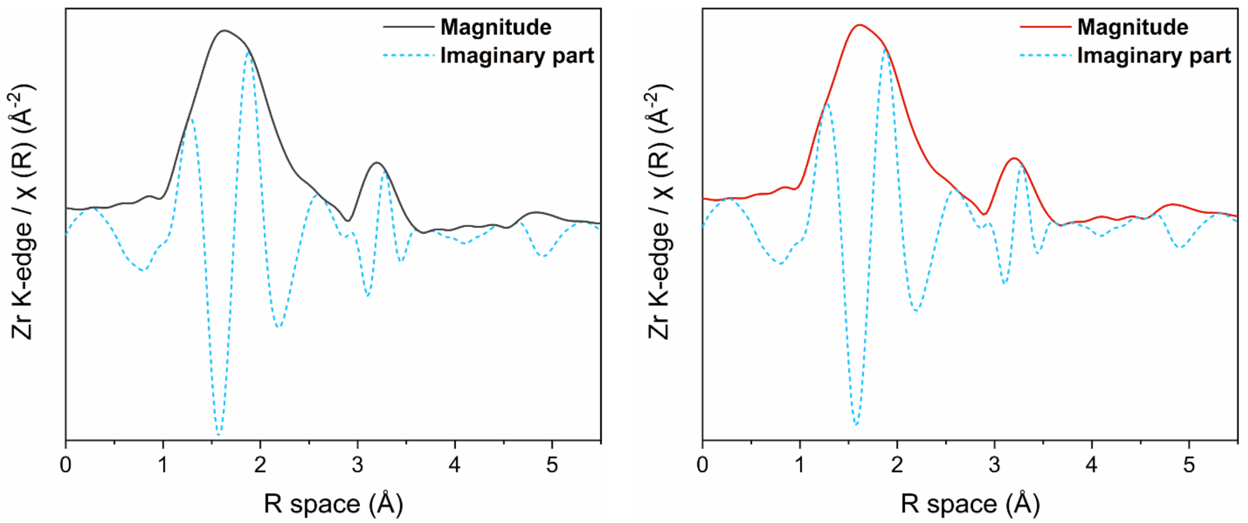

Figure S33. *k*-weighted χ(r) (solid) and Img [χ(r)] (dashed) EXAFS spectra of MIP-206-OH before (left) and after PFOA capture (right).

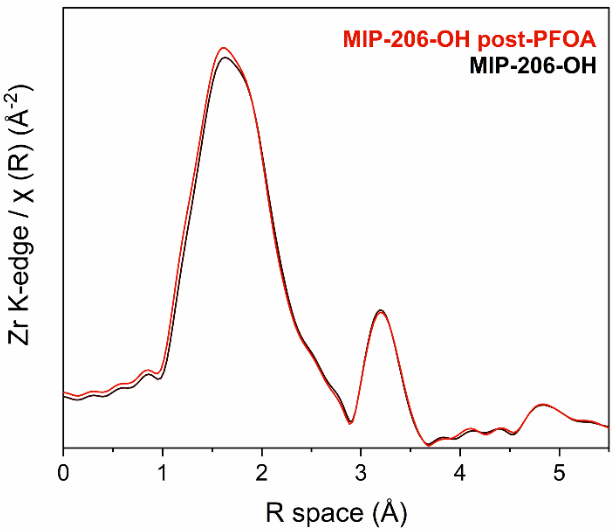

Figure S34. EXAFS spectra comparison (Zr *K*-edge) of MIP-206-OH before and after capture of PFOA.

S6. X-Ray Pair Distribution Function analysis

Synchrotron X-ray total scattering data suitable for pair distribution function (PDF) analysis were collected at Diamond Light Source, UK (beamline I15-1, beamtime CY37864-1 using 76.6 keV (0.161669 Å) X-rays. Samples were first ground into fine powder, then loaded into kapton capillaries (1 mm Ø) and sealed. Data scans were collected for 10 minutes. Empty capillary and background total scattering data were also collected for in data processing. Sample data were processed using PDFgetX3 to a Qmax of 22 Å^{-1}. In all cases, differential PDFs were obtained by subtraction of PDF profiles in real space after applying a normalization factor.

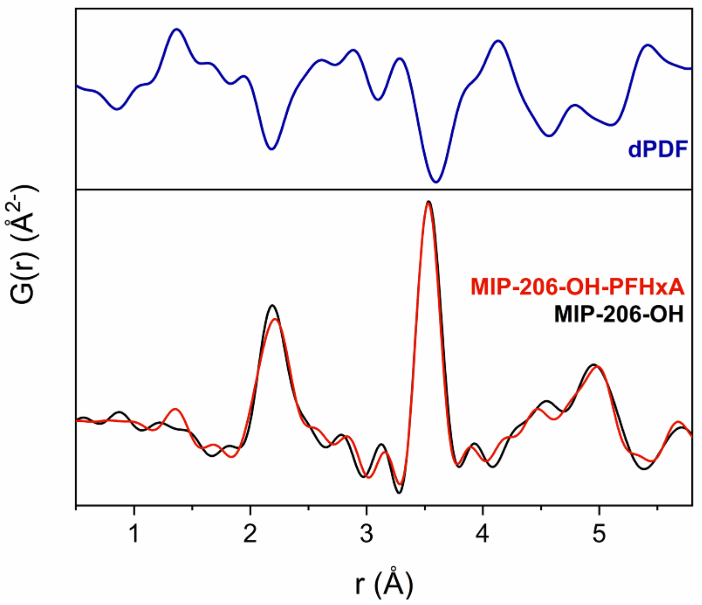

Figure S35. PDF data of MIP-206-OH before and after PFHxA treatment. The corresponding dPDF signal (blue) shows the same PFAS-related peaks detailed in the main text.

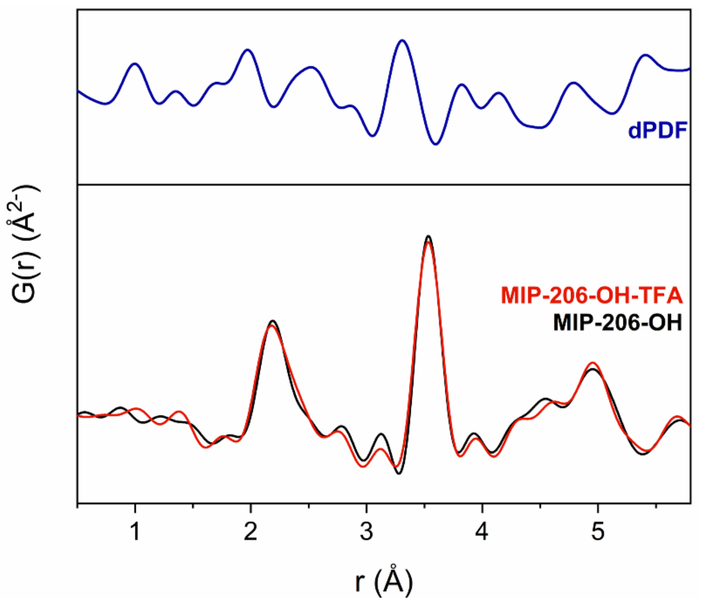

Figure S36. PDF data of MIP-206-OH before and after TFA treatment. The corresponding dPDF signal (blue) shows the same PFAS-related peaks detailed in the main text, however with different relative intensities due to the different number of interatomic distances related to the short-chain TFA in comparison to longer-chain PFAS.

S7. Supplementary References

1. Loukopoulos, E.; Barragán-Soto, S.; Marugán-Benito, S.; Borrego-Marin, E.; Navarro, J. A. R.; Platero-Prats, A. E., Enhanced CO2 affinity in a metal–organic framework through green incorporation of a dual-functional amino acid. *Nanoscale* **2026,** *18,* 706-711.

2. Macrae, C. F.; Edgington, P. R.; McCabe, P.; Pidcock, E.; Shields, G. P.; Taylor, R.; Towler, M.; van De Streek, J., Mercury: visualization and analysis of crystal structures. *J. Appl. Crystallogr.* **2006,** *39*, 453-457.

3. Wang, S.; Chen, L.; Wahiduzzaman, M.; Tissot, A.; Zhou, L.; Ibarra, I. A.; Gutiérrez-Alejandre, A.; Lee, J. S.; Chang, J. S.; Liu, Z.; Marrot, J.; Shepard, W.; Maurin, G.; Xu, Q.; Serre, C., A Mesoporous Zirconium-Isophthalate Multifunctional Platform. *Matter* **2021,** *4*, 182-194.

4. Bon, V.; Senkovska, I.; Baburin, I. A.; Kaskel, S., Zr- and Hf-based metal-organic frameworks: Tracking down the polymorphism. *Crystal Growth and Design* **2013,** *13*, 1231-1237.

5. Vanoursouw, T. M.; Rottiger, T.; Wadzinski, K. A.; Vanderwaal, B. E.; Snyder, M. J.; Bittner, R. T.; Farha, O. K.; Riha, S. C.; Mondloch, J. E., Adsorption of a PFAS Utilizing MOF-808: Development of an Undergraduate Laboratory Experiment in a Capstone Course. *Journal of Chemical Education* **2023,** *100*, 861-868.

6. Nassazzi, W.; Lai, F. Y.; Ahrens, L., A novel method for extraction, clean-up and analysis of per- and polyfluoroalkyl substances (PFAS) in different plant matrices using LC-MS/MS. *J. Chromatogr. B* **2022,** *1212*, 123514.

7. Li, R.; Alomari, S.; Stanton, R.; Wasson, M. C.; Islamoglu, T.; Farha, O. K.; Holsen, T. M.; Thagard, S. M.; Trivedi, D. J.; Wriedt, M., Efficient Removal of Per- And Polyfluoroalkyl Substances from Water with Zirconium-Based Metal-Organic Frameworks. *Chem Mater* **2021,** *33*, 3276-3285.

8. Sini, K.; Bourgeois, D.; Idouhar, M.; Carboni, M.; Meyer, D., Metal-organic framework sorbents for the removal of perfluorinated compounds in an aqueous environment. *New Journal of Chemistry* **2018,** *42*, 17889-17894.

9. Sini, K.; Bourgeois, D.; Idouhar, M.; Carboni, M.; Meyer, D., Metal-organic frameworks cavity size effect on the extraction of organic pollutants. *Materials Letters* **2019,** *250,* 92-95.

10. Liu, K.; Zhang, S.; Hu, X.; Zhang, K.; Roy, A.; Yu, G., Understanding the Adsorption of PFOA on MIL-101(Cr)-Based Anionic-Exchange Metal-Organic Frameworks: Comparing DFT Calculations with Aqueous Sorption Experiments. *Environ Sci Technol* **2015,** *49,* 8657-65.

11. Luo, J.; Luo, F.; Li, H.; Mao, C.; Pan, Y.; Fang, Z.; Yu, D.; Liu, H.; Fu, K., Structure-Oriented Metal-Organic Framework Activation via Proximal Oligoalkyl Quaternary Ammonium Grafting Enhances Long-Chain PFAS Sorption. *Angew. Chem. Int. Ed.* **2025**, e202514746.

12. Liang, R. R.; Xu, S.; Han, Z.; Yang, Y.; Wang, K. Y.; Huang, Z.; Rushlow, J.; Cai, P.; Samorì, P.; Zhou, H. C., Exceptionally High Perfluorooctanoic Acid Uptake in Water by a Zirconium-Based Metal-Organic Framework through Synergistic Chemical and Physical Adsorption. *J. Am. Chem. Soc.* **2024,** *146*, 9811-9818.

13. Hedbom, D.; Gaiser, P.; Günther, T.; Cheung, O.; Strømme, M.; Åhlén, M.; Sjödin, M., A fluorinated zirconium-based metal-organic framework as a platform for the capture and removal of perfluorinated pollutants from air and water. *J. Mater. Chem. A* **2024,** *13*, 1731-1737.

14. Loukopoulos, E.; Marugán-Benito, S.; Raptis, D.; Tylianakis, E.; Froudakis, G. E.; Mavrandonakis, A.; Platero-Prats, A. E., Chemically Tailored Metal-Organic Frameworks for Enhanced Capture of Short- and Long-Chain Per- and Polyfluoroalkyl Substances from Water. *Adv. Funct. Mater.* **2024,** *34*, 2409932.

15. Dalapati, R.; Shi, J.; Hunter, M.; Zang, L., Dual-functional metal-organic framework for efficient removal and fluorescent detection of perfluorooctanoic acid (PFOA) from water. *Journal of Materials Chemistry C* **2025,** *13*, 16753-16762.

16. Liang, R. R.; Yang, Y.; Han, Z.; Bakhmutov, V. I.; Rushlow, J.; Fu, Y.; Wang, K. Y.; Zhou, H. C., Zirconium-Based Metal–Organic Frameworks with Free Hydroxy Groups for Enhanced Perfluorooctanoic Acid Uptake in Water. *Adv. Mater.* **2024,** *36*, 2407194.

17. Yang, Y.; Zheng, Z.; Ji, W.; Xu, J.; Zhang, X., Insights to perfluorooctanoic acid adsorption micro-mechanism over Fe-based metal organic frameworks: Combining computational calculation with response surface methodology. *Journal of Hazardous Materials* **2020,** *395*.

18. Mohd Azmi, L. H.; Williams, D. R.; Ladewig, B. P., Polymer-assisted modification of metal-organic framework MIL-96 (Al): influence of HPAM concentration on particle size, crystal morphology and removal of harmful environmental pollutant PFOA. *Chemosphere* **2021,** *262*.

19. Ravel, B.; Newville, M., ATHENA, ARTEMIS, HEPHAESTUS: Data analysis for X-ray absorption spectroscopy using IFEFFIT. *Journal of Synchrotron Radiation* **2005,** *12*, 537-541.
